# The effect of age on DNA methylation in whole blood among Bangladeshi men and women

**DOI:** 10.1186/s12864-019-6039-9

**Published:** 2019-09-10

**Authors:** Rick J. Jansen, Lin Tong, Maria Argos, Farzana Jasmine, Muhammad Rakibuz-Zaman, Golam Sarwar, Md. Tariqul Islam, Hasan Shahriar, Tariqul Islam, Mahfuzar Rahman, Md. Yunus, Muhammad G. Kibriya, John A. Baron, Habibul Ahsan, Brandon L. Pierce

**Affiliations:** 1 Department of Public Health, North Dakota State University, Fargo, ND USA; 2 Genomics and Bioinformatics Program, North Dakota State University, Fargo, ND USA; 3 Biostatistics Core Facility, North Dakota State University, Fargo, ND USA; 4 Department of Public Health Sciences, University of Chicago, 5841 S. Maryland Ave., W264, MC2000, Chicago, IL 60637 USA; 5 Divison of Epidemiology and Biostatistics, School of Public Health, University of Illinois at Chicago, Chicago, IL USA; 6 UChicago Research Bangladesh Mohakhali, Dhaka, 1230 Bangladesh; 7 Research and Evaluation Division BRAC, Mohakhali, Dhaka, 1212 Bangladesh; 8 International Centre for Diarrhoeal Disease Research Bangladesh, Dhaka, 1000 Bangladesh; 9 Department of Epidemiology, Gillings School of Global Public Health, University of North Caroline, Chapel Hill, NC USA; 10 Department of Medicine, The University of Chicago, Chicago, IL USA; 11 Department of Human Genetics and Comprehensive Cancer Center, The University of Chicago, Chicago, IL USA

**Keywords:** Genome-wide, Age-associated, Methylation, Sex-specific, Cell type adjustment, RefFreeEWAS, Age prediction model

## Abstract

**Background:**

It is well-known that methylation changes occur as humans age, however, understanding how age-related changes in DNA methylation vary by sex is lacking. In this study, we characterize the effect of age on DNA methylation in a sex-specific manner and determine if these effects vary by genomic context. We used the Illumina HumanMethylation 450 K array and DNA derived from whole blood for 400 adult participants (189 males and 211 females) from Bangladesh to identify age-associated CpG sites and regions and characterize the location of these age-associated sites with respect to CpG islands (vs. shore, shelf, or open sea) and gene regions (vs. intergenic). We conducted a genome-wide search for age-associated CpG sites (among 423,604 sites) using a reference-free approach to adjust for cell type composition (the R package RefFreeEWAS) and performed an independent replication analysis of age-associated CpGs.

**Results:**

The number of age-associated CpGs (*p* < 5 x 10^− 8^) were 986 among men and 3479 among women of which 2027(63.8%) and 572 (64.1%) replicated (using Bonferroni adjusted *p* < 1.2 × 10^− 5^). For both sexes, age-associated CpG sites were more likely to be hyper-methylated with increasing age (compared to hypo-methylated) and were enriched in CpG islands and promoter regions compared with other locations and all CpGs on the array. Although we observed strong correlation between chronological age and previously-developed epigenetic age models (*r* ≈ 0.8), among our top (based on lowest *p*-value) age-associated CpG sites only 12 for males and 44 for females are included in these prediction models, and the median chronological age compared to predicted age was 44 vs. 51.7 in males and 45 vs. 52.1 in females.

**Conclusions:**

Our results describe genome-wide features of age-related changes in DNA methylation. The observed associations between age and methylation were generally consistent for both sexes, although the associations tended to be stronger among women. Our population may have unique age-related methylation changes that are not captured in the established methylation-based age prediction model we used, which was developed to be non-tissue-specific.

**Electronic supplementary material:**

The online version of this article (10.1186/s12864-019-6039-9) contains supplementary material, which is available to authorized users.

## Background

The epigenome is believed to have significant plasticity throughout life and is likely influenced by a variety of factors including diet, inflammation, physical activity, smoking, and aging [1, 2]. DNA (deoxyribonucleic acid) methylation at CpG (5′—C—phosphate—G—3′) sites (DNA regions where a guanine nucleotide follows a cytosine) is the most commonly studied epigenetic feature in human populations. DNA methylation patterns are known to be tissue specific, although some CpGs show similar methylation levels across tissues [3–6]. Methylation patterns of DNA extracted from blood have been associated with gender [7–9], aging [10–18], embryonic growth restriction [19], and many age-related diseases, such as cancer and diabetes [20–23]. Additionally, variation in DNA methylation has been suggested to explain disease phenotype differences between monozygotic twins [24–27] and associations between in utero environment and diseases during adult life [28, 29]. Mechanistically, variation in DNA methylation likely reflects variation in histone modifications, chromatin conformation, and gene expression [30], with hypo-methylation of the promoter region and hyper-methylation of the gene body often reflecting increased expression [31].

Alterations in DNA methylation that occur as humans age have been described [10, 11, 14, 32–35]. Analysis of genome-wide DNA methylation in blood cells has demonstrated that 15–30% of CpG sites are associated with age [36–38]. In addition, DNA methylation has been used as a measure of “epigenetic aging” (i.e., epigenetic clock) and to investigate potential environmental factors that affect biological aging [38–41]. An accelerated epigenetic clock has been associated with higher mortality risk, as well as reduced cognitive and physical health [42, 43].

Prior studies have conducted genome-wide searches for age-associated CpG sites in humans. Most have been conducted using data from individuals of European ancestry, and none have done so in a sex-specific manner [44]. In this study, we used genome-wide methylation data on 189 males and 211 females from Bangladesh to identify age-associated CpG sites in a sex-specific manner and characterize these CpG sites with respect to genomic context. We chose to conduct a stratified analysis as there are many biological differences between males and females that may impact how the epigenome changes with age. Understanding how methylation changes with age is critical for understanding biological processes associated with human aging and the role of epigenetics in susceptibility to aging-related diseases.

## Methods

### Study sample

The Bangladesh Vitamin E and Selenium Trial (BEST) is a 2 × 2 factorial randomized chemoprevention trial evaluating the long-term effects of vitamin E and selenium supplementation on non-melanoma skin cancer risk and has been described in detail elsewhere [45]. Participants were eligible for BEST if they resided in select rural communities in central Bangladesh, were between ages 25 and 65 years old, had arsenic-induced skin lesions, and no prior cancer history. Between April 2006 and August 2009, a total of 7000 individuals were enrolled. In-person interviews, clinical evaluations, and urine and blood sample collection were performed by trained study physicians, blinded to participants’ arsenic exposure using structured protocols. For the present study, 413 participants with baseline specimens collected prior to the intervention were randomly sampled.

The study protocol was approved by the relevant institutional review boards in the United States (The University of Chicago and Columbia University) and Bangladesh (Bangladesh Medical Research Council). Informed consent was provided by participants prior to the original BEST study.

### Measurement of methylation

Details on methylation measurement in this population have been given in detail elsewhere [46]. Briefly, DNA was extracted using DNeasy Blood kits (Qiagen, Valencia, CA, USA), and bisulfite conversion was performed using the EZ DNA Methylation Kit (Zymo Research, Irvine, CA, USA). DNA methylation was measured in 500 ng of bisulfite-converted DNA per sample using the Illumina HumanMethylation 450 K (485,577 CpG sites) BeadChip kit (Illumina, San Diego, CA, USA) according to the manufacturer’s protocol. The average methylation at each CpG site is represented as a continuous score (β value) between 0 (unmethylated) and 1 (completely methylated). From the 413 participants, we excluded 6 samples for inconsistency between self-reported and methylation-derived sex, and 7 samples with > 5% of CpGs either having p for detection > 0.05 or missing values. This resulted in 400 samples used for analyses (189 males and 211 females). We excluded 416 probes on the Y chromosome, probes lacking chromosome data (mostly control probes; *n* = 65), probes mapping to multiple locations (*n* = 41,937), probes with target CpG sites containing SNPs (*n* = 20,869), and probes with > 10% missing data across samples (*n* = 1932). This resulted in a total of 423,188 probes included in this analysis. Based on 11 samples run in duplicate across two different plates, the average inter-assay Spearman correlation coefficient was 0.987 (range, 0.974–0.993).

### Measurement of gene expression

Sample processing for gene expression analysis has been described previously in detail [46]. Briefly, RNA (ribonucleic acid) was extracted from stored Mononuclear cells using RNeasy Micro Kit from QIAGEN (Valencia, CA, USA). Nanodrop 1000 spectro-photometer (Thermo Scientific, Wilmington, DE, USA) was used to check RNA concentration and quality and the Illumina TotalPrep 96 RNA Amplification kit was used for cDNA synthesis. The Illumina HumanHT-12-v4 BeadChip (47,231 probes covering 31,335 genes) was used to measure transcript abundance according to manufacturer’s protocol.

### Statistical analysis

For each CpG site, a sex-stratified linear regression model was used to assess the association between age in years (independent variable) and the logit-transformed methylation β value (ratio of methylated to unmethylated alleles; dependent variable). Coefficients and standard errors (SEs) from the regression models correspond to a 1-year age increase. To increase our chances of finding truly significant results and account for multiple testing in both sex-specific models, we use a significance threshold (*p* < 5 × 10^− 8^) slightly more stringent than the Bonferroni-corrected value (*p* < 6 × 10^− 8^ = 0.05/(423,188*2)). For differentially methylated probes with *p* < 5 × 10^− 8^, we used sex-stratified linear regressions to examine the association of methylation with corresponding RNA transcript levels of the gene assigned to the methylation locus (based on Illumina’s annotation file). To control for the potential confounder, cell type composition, we used the RefFreeEWAS method [47]. In a separate analysis, we used the reference-based method, MethylSpectrum [48], a reference-based adjustment for blood cell type, but the resulting volcano plot (not shown) was asymmetric toward hyper-methylation of sites; potentially representing the effects of unmeasured confounding. Therefore, we present results from the analysis using the RefFreeEWAS method. This method empirically establishes the top *d* number (user setting; we used d = 5) of latent variables for which to adjust. An additional covariate in our models adjusted for batch (or plating) effect. For the enrichment analyses, we used a Fisher exact test to determine if a higher proportion of significant (*p* < 0.05) CpGs were found in a specific genomic region compared to all analyzed CpGs. We conducted a second set of tests to compare the number of significant CpGs found within a specific genome region between male and female to see if there was a difference by sex. Among the top 100 CpGs within each sex, we used a linear regression model with logit-transformed CpG beta values and cell type composition matrix (set of 6 blood cell type variables estimated using methyl spectrum) as the independent variables and the expression levels for the Illumina assigned gene as the dependent variable to identify significant methylation-expression associations. For those CpG-gene expression sets found to have a significant association, we reran the regression model with an additional age and age-CpG interaction term. A Bonferroni corrected *p* (males: 0.05/417 and females: 0.05/538) was considered to be statistically significant. We used the R Statistical package v3.2.5 [49] to run all analyses.

## Results

### Demographics

Comparing participant characteristics by sex (Table 1), we observed significant differences among most variables. On average, males had a higher proportion who smoked and a higher proportion of T-helper (CD4T) cells. A lower proportion of males compared to females had high urinary arsenic levels and had a lower proportion of circulating natural killer (NK), monocytes (Mono), and granulocytes (Gran) cells. The mean age was 43.1 (standard deviation (SD) = 9.1) for males and 44.3 (SD = 11.1) for females and there was a significant difference in the age distribution between sexes (Additional file 1).

**Table 1:** Characteristics of study participants, by sex

	Males (*N* = 189)		Females (*N* = 211)		
	N	%	N	%	*P* value*
Age					0.0099*
<35	36	19.0	50	23.7	
≥35-<45	61	32.3	53	25.1	
≥45-<55	67	35.4	57	27.0	
≥55	25	13.2	51	24.2	
BMI					0.2527*
<18.5	65	34.4	87	41.2	
≥18.5-<24.5	99	52.4	106	50.2	
≥24.5	25	13.2	18	8.5	
Smoking					< 0.0001*
Never	7	3.7	102	48.3	
Ever	182	96.3	109	51.7	
Urinary Total Arsenic Concentration (μg/g)					0.0004*
<15	62	32.8	36	17.1	
≥15 - < 37.5	48	25.4	54	25.6	
≥37.5 - < 104.2	46	24.3	54	25.6	
≥104.2	33	17.5	67	31.8	
	mean	range	mean	range	*P* value*
Average Blood Cell Percentages (estimated by MethylSpectrum)					
CD8T	0.22	0.07–0.38	0.2	0.05–0.42	0.0437*
CD4T	0.07	0–0.25	0.05	0–0.23	0.0016*
NK	0.02	0–0.09	0.03	0–0.09	< 0.0001*
Bcell	0.08	0.03–0.20	0.08	0.02–0.15	0.8230*
Mono	0.07	0–0.14	0.08	0–0.15	0.0046*
Gran	0.48	0.34–0.63	0.49	0.31–0.70	0.0909*

*Abreviations*: *N* Number, % Percent

*Chi-squared test used for categorical variable and t-test used for continuous variables

### Sex-specific age-associated CpG sites

**Fig. 1:**
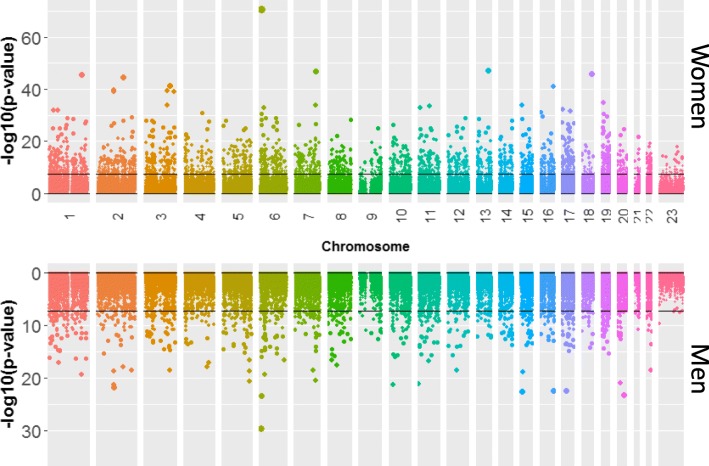
Manhattan plot showing the association between age and CpG sites for 211 women (top) and 189 men (bottom). Horizontal black line indicates *p* =5 × 10^− 8^

At *p* threshold of 5 × 10^− 8^, we observed 3479 CpG sites at which methylation was associated with age among women and 986 among men (Fig. 1). Focusing only on these significant sites, there is some overlap between the sexes (530 in common between women and men significant sets). However, among the 3479 age-associated methylation sites among women, 3048 (87.6%) are age-associated methylation sites among men at a *p* < 0.05 and likewise, among the 986 age-associated methylation sites among men, 946 (95.9%) are age-associated methylation sites among women at a *p* < 0.05. The 50 most significant CpGs for each sex are reported in Additional file 2 with 32 age-associated CpGs in common among the top 100 male and female RefFreeEWAS results (Additional file 3). Additional file 4 shows a comparison between several RefFreeEWAS models some of which are sex-specific and some adjust for smoking. Interestingly, the overlap in top 100 CpGs when comparing male only to female only models is 31; while the same comparison for models that adjust for smoking produces only 28 overlapping CpGs.

We used an independent validation set consisting of 400 Bangladeshi individuals (167 males) participating in the Health Effects of Arsenic Longitudinal Study (HEALS) [50] to assess overlap of significant age-associated CpGs identified in this current study. In this sample 90% of females reported never smoking compared with 74% of males reporting ever smoked. The mean age was 41.1 (SD = 10.1) for males and 34.4 (SD = 8.8) for females with a significant difference in the age distribution between sexes (Additional file 5). For BEST the 450 K (CpGs) Illumina chip was used while the EPIC array (~ 850 K CpGs) was used in HEALS. Because 47,780/423604 (11.3%) CpGs where not present on the 850 K chip, we were unable to validate observed significant results for 93/986 (9.4%) CpGs among males, 301/3479 (8.7%) among females, and 9/100 (9%) of the top 100 CpGs among both sexes. Using the 3178 overlapping age-associated CpGs observed as significant (*p* < 5 × 10^− 8^) among females in BEST, 2027(63.8%) (using bonferroni adjusted *p* < 1.2 × 10^− 5^) were also significantly associated with age in HEALS. Likewise for males, among the 893 overlapping and significant (*p* < 5 × 10^− 8^) age-associated CpGs observed in BEST, 572 (64.1%) (using bonferroni adjusted *p* < 1.2 × 10^− 5^) were also significantly associated with age in HEALS. In the model adjusting for smoking status, the corresponding numbers and percentages among females were 1781/3294 or 54.1% and among males were 449/716 or 62.7%.

Additional file 6 shows the beta values and *p*-values for the top 100 age-associated CpGs identified in BEST of which 68/91 (74.7%) among males and 81/91 (89.0%) among females are significantly associated with age in HEALS using a *p* < 5 × 10^− 8^ while all overlapping CpGs are significant at *p* < 0.05 among both sexes. Overlapping number of CpGs across additional sex stratified and sex adjusted models and significant sets can be observed in the Additional file 7a and b.

**Fig. 2:**
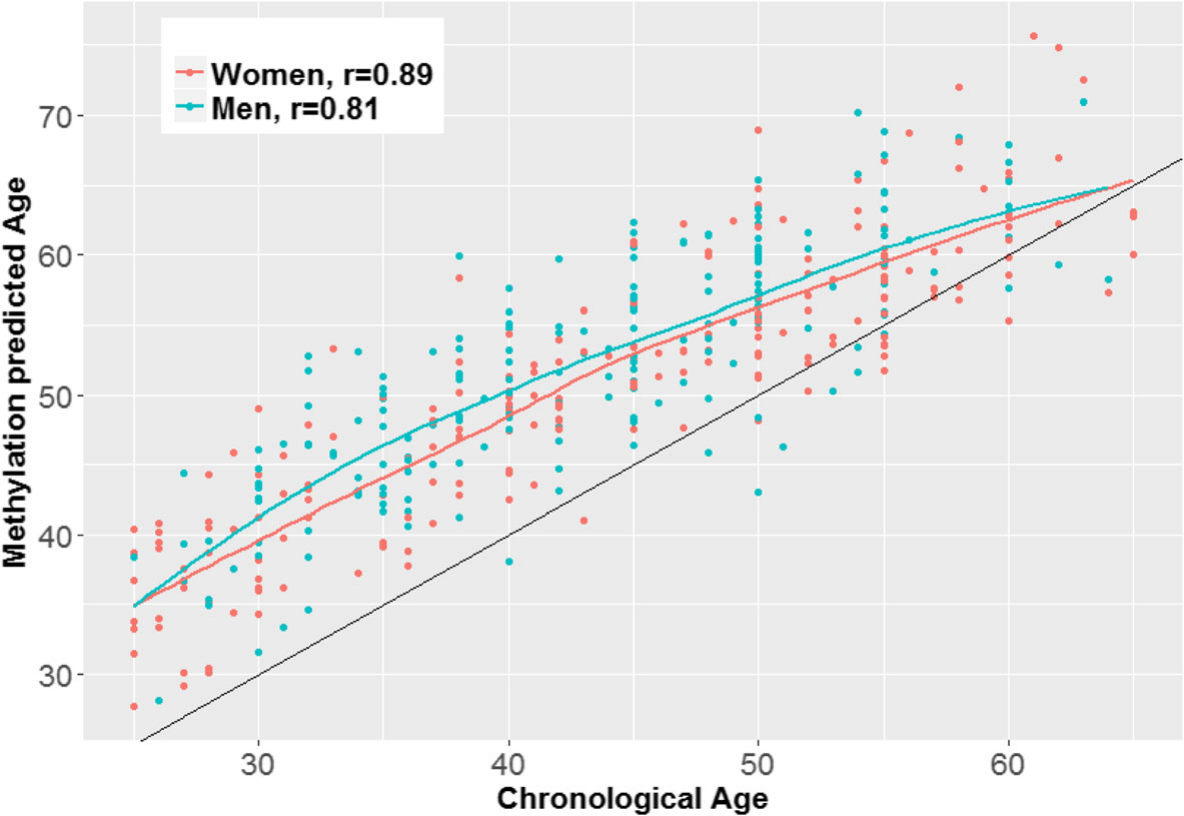
Correlation between chronological age and predicated age using Horvath [40] identified age-related CpG markers among 211 women and 189 men. Colored lines in the plot represent loess lines of best fit and the black line represents perfect correlation

We examined associations with age for the 354 CpGs included in the Horvath methylation age predictor [40] . The predicted age based on the calculator showed a strong correlation (r) with chronological age among both women (*r* = 0.89) and men (*r* = 0.81) (Fig. 2). While only 12 of our age-associated methylated loci among men and 44 among women were included in the 354 Horvath CpGs (Additional file 3), 140 of the Horvath CpG sites were differentially methylated among women in the expected direction (*p* < 0.05), while 111 were differentially methylated among men (*p* < 0.05 and expected direction). The median chronological age was younger for both sexes compared to the predicted methylation age and was 44 vs. 51.7 in males and 45 vs. 52.1 in females. Potential reasons for this discrepancy are mentioned in the discussion section.

### Characterization age-associated CpG sites with respect to genomic region

**Fig. 3:**
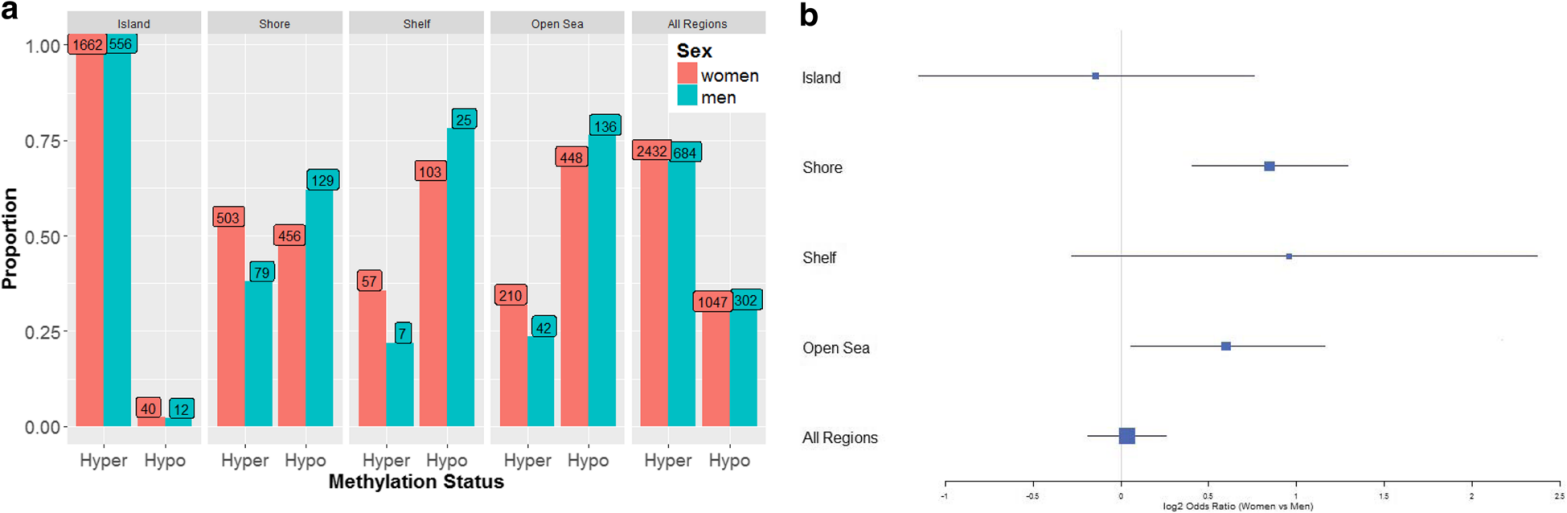
a) Proportion of hyper-methylation vs. hypo-methylation among age-associated CpGs (*p* < 5 x 10^− 8^) by genomic region, stratified by sex. b) Log2 odds ratio using the median unbiased estimate and the mid-p exact 95% confidence interval were used to compare women to men by genomic region in aggregate table format. All Fisher exact tests comparing the proportiontion of hyper-methylation (vs. hypo-methylation) within individual categories to "all regions" were significant among either men or women with all *p* < 4.5 x 10^− 8^. For Fisher exact tests comparing the proportion of hyper-methylation (vs. hypomethlylation) between men and women within each category, only shore and open sea where significant with *p* = 0.0001 and 0.0342, respectively

**Fig. 4:**
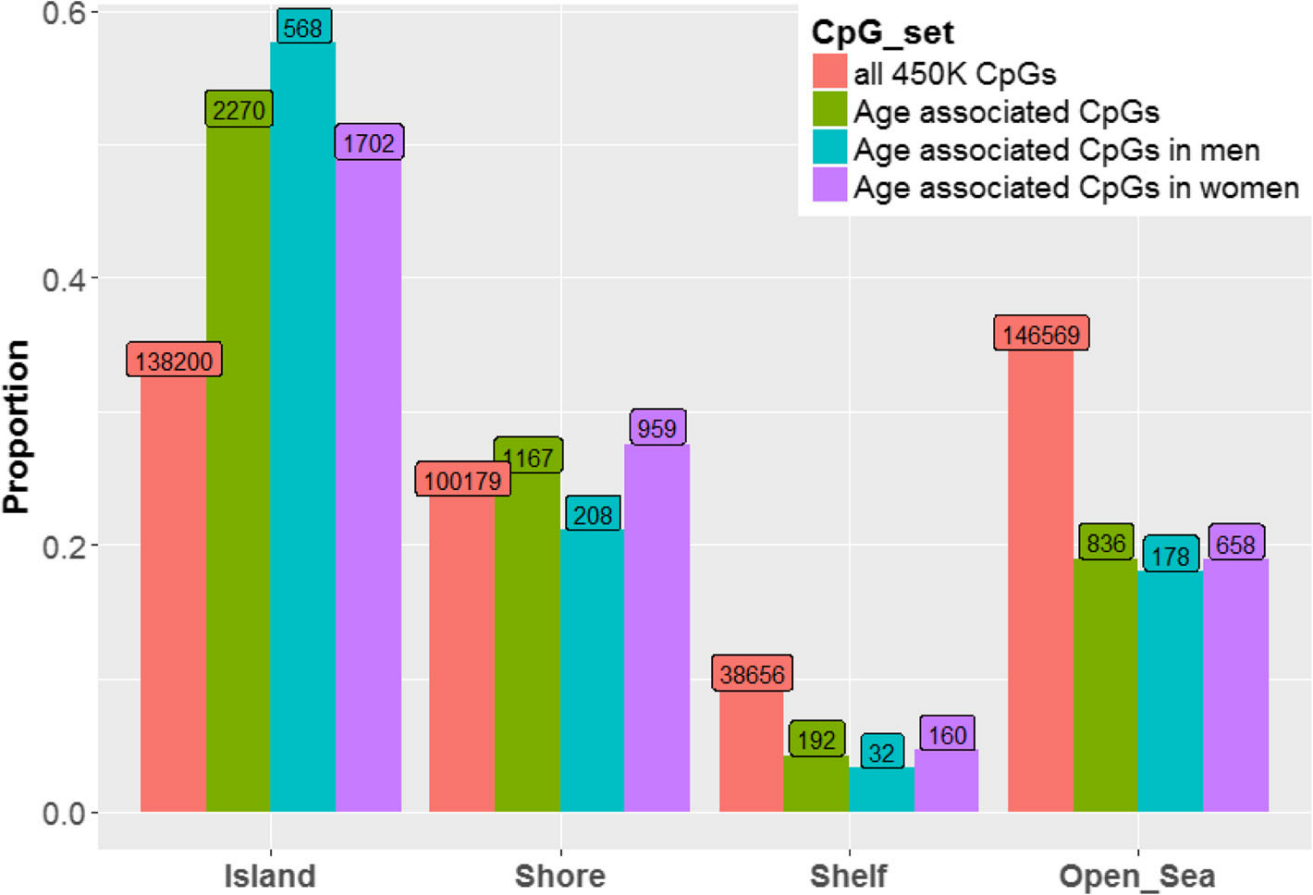
Proportion of CpGs residing in genomic regions defined by CpG density. All but the red bar represents age-associated CpGs. Fisher exact tests comparing enrichment/depletion within individual categories to all categories were significant (*p* < 2 x 10^-11^) among men for island, shelf, and open sea regions and among women for all four categories (*p* = 3.8 x 10^-5^). Fisher exact tests comparing enrichment/depletion within categories between men and women were significant for island (*p* = 0.0071) and shore (*p* = 0.0015) regions

In order to determine if proximity to CpG islands was related to age-related differences in CpG methylation, we used categories defined by Illumina (i.e., island, shore, shelf, open sea) to estimate the proportion of age-associated CpGs that were hyper- vs. hypo-methylated within each category (Fig. 3). Across all CpGs, we observed a higher proportion of hyper-methylated vs. hypo-methylated sites, with a > 2-fold difference for both sexes. This difference was largely driven by CpGs in island regions, which were almost exclusively hyper-methylated with increasing age, with > 97% of CpGs showing hyper-methylation among both sexes. In contrast, in shelf and open sea regions, there were substantially more age-associated CpGs that were hypometylated with increasing age among both sexes (Fig. 3). Shore regions had a higher proportion of hyper-methylation among women, but higher proportion of hypo-methylation among men (Fisher exact test *p* = 0.0001). We also wanted to determine if age-associated CpGs were enriched in any of these categories. Compared to all CpG probes analyzed, age-associated CpG sites were strongly (all test *p* < 1 x 10^− 11^) enriched in island regions and depleted in shelf and open sea regions (Fig. 4). Approximately two-fold enrichment/depletion was observed in these categories. We found evidence for slight enrichment in shore regions among women only (*p* = 3.8 x 10^-5^).

### Characterization the top CpG sites for each sex in relationship to gene location

**Fig. 5:**
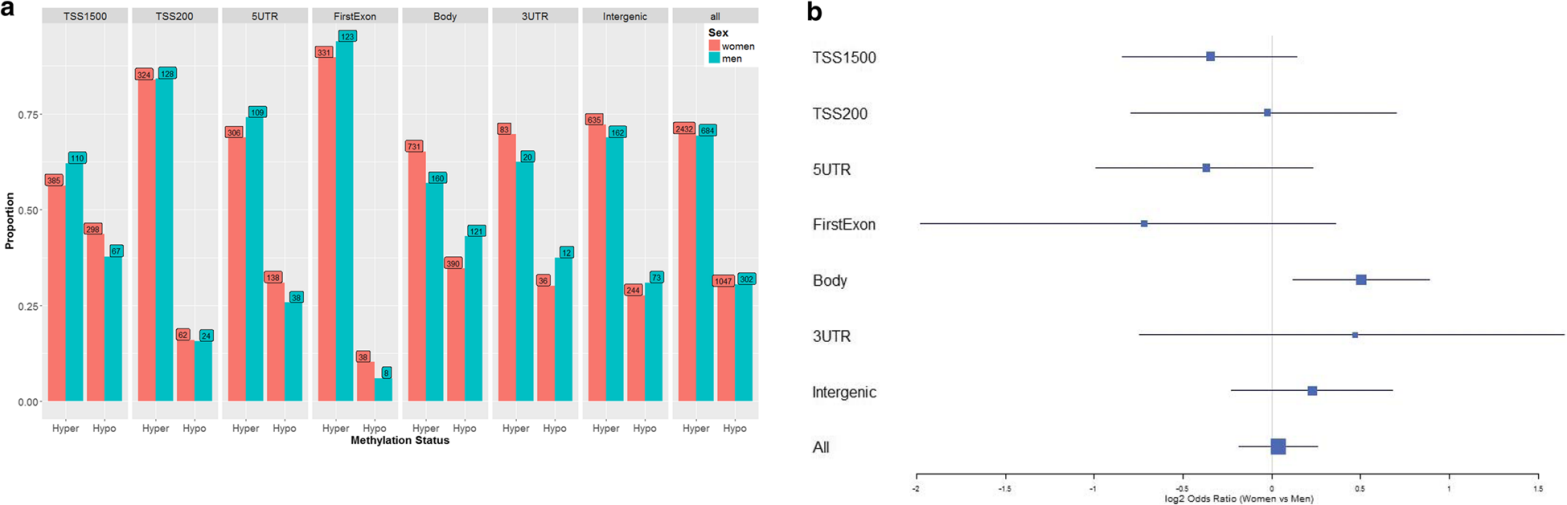
a) Proportion of hyper-methylated and hypo-methylated CpGs among age-associated CpGs (*p* < 5 x 10^− 8^) by relationship to gene category, stratified by sex. b) Log2 odds ratio using the median unbiased estimate and mid-p exact 95% confidence interval were used to compare women to men within each category in aggregate table format. Fisher exact tests comparing the proportion of hyper-methylation (vs. hypo-methylation) within individual categories to all categories were significant among men for TSS200 (*p* = 0.0001), first exon (*p* = 7.3 x 10^-11^), and body (*p* = 0.0001) regions and among women for TSS1500 (*p* = 1.2 x 10^-11^), TSS200 (*p* = 1.6 x 10^-9^), first exon (*p* = 2.2 x 10^-16^), and body (*p* = 0.0034) regions. For Fisher exact tests comparing hyper-methylation between men and women within each category, only body (*p* = 0.0125) was significant

In order to determine if proximity to genes was related to methylation at age-related CpGs, we examined the proportion of hyper- vs. hypo-methylation at age-related CpGs within categories defined by Illumina (i.e., within 1500 basepairs (bp) of a transcription start site (TSS1500), within 200 bp of a TSS (TSS200), in a 5′ untranslated region (UTR), in the first exon, in the gene body, in the 3′ UTR, and Intergenic). In all categories, the proportion of age-associated CpGs that were hyper-methylated was greater than the hypo-methylated proportion (Fig. 5). This difference was most pronounced in the first exon and the TSS200 categories (*p* < 0.0001 for both categories, in both sexes). The gene body category showed evidence of depleted for hyper-methylated sites in both sexes (*p* < 0.005), when compared to all sites.

**Fig. 6:**
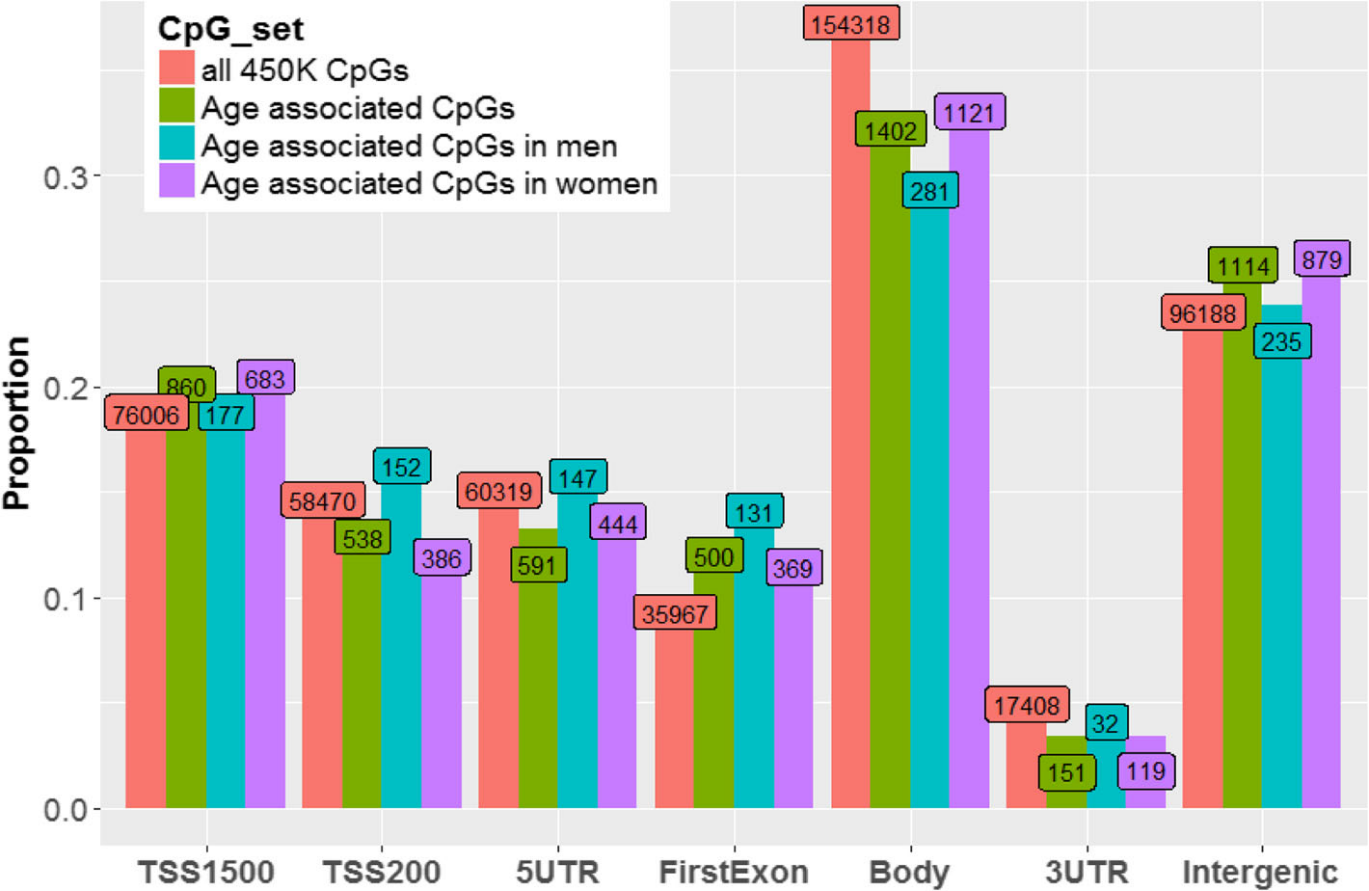
Proportion of age-associated CpGs residing in regions defined by gene features. The red bar represents all measured CpGs. Fisher exact tests comparing enrichment/depletion within individual categories to all categories were significant among men for first exon (*p* = 5.7 x 10^-6^) and body (*p* = 0.0002) regions and among women for TSS1500 (*p* = 0.0337), TSS200 (*p* = 3.5 x 10^-5^), 5′ UTR (*p* = 0.0307), first exon (*p* = 7.9 x 10^-5^), body (*p* = 0.0003), and intergenic (*p* = 0.0054). Fisher exact tests comparing enrichment/depletion within individual categories between men and women were significant for TSS200 (*p* = 0.0016) and first exon (*p* = 0.0419)

We examined the proportions of age-associated CpGs in each category, and observed that enrichment/depletion compared to all 450 K CpGs varied across categories with the strongest enrichment occurring in the first exon category (*p* < 5 x 10^-5^) and the strongest depletion occurring in the gene body category (*p* < 0.0003) (Fig. 6). While the observed enrichment/depletion features appeared to be quite consistent across sexes, a slightly higher proportion of the age-associated CpGs were observed among men in the TSS200 (*p* = 0.0016) and first exon (*p* = 0.0419) locations.

### Expression of genes assigned to top 100 age-associated CpG sites

**Fig. 7:**
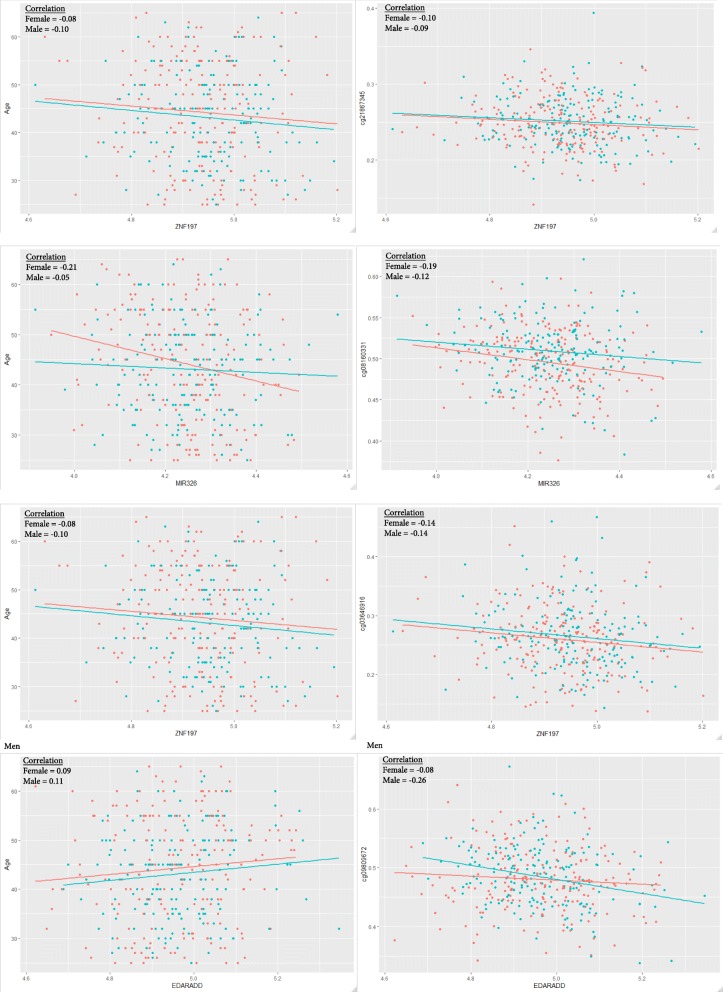
Scatterplots of CpG beta values and the expression of its Illumina-assigned gene (right-hand side) and scatterplots of the expression of the same Illumina-assigned gene by age (left side). CpGs and gene pairs were chosen based on significant age interactions within the regression model. All plots distinguish points as female (salmon color) or male (blue color) and include a linear line of best fit for each sex

In an attempt to understand the potential gene-regulatory implications of the top 100 (lowest *p*-values) age-associated CpGs within each sex, we estimated the association using a regression model between our top age-associated CpGs and expression values for the gene assigned (by Illumina) to each CpG along with all genes in the region +/− 200 basepairs around each CpG. Among the 100 top age-associated CpGs in each set, there were 417 CpG-gene associations tested among males and 538 associations tested among females. Among the 417 in the male set, we observed 3 significant (*p* < 0.0001) associations between methylation and expression; 2 of these (66%) were inverse associations. Among the 538 in the female set, 11 showed significant associations (*p* < 0.00009), and 8(73%) were inverse. Based on these significant associations, we looked for evidence that a CpG-expression relationship varied with age by adding an age interaction term to the regression model which may suggest there are functional changes to the way the CpG and gene expression associate with age. We observed 1/3 significant interactions with age among males and 3/11 among females and show the age-gene expression and CpG-gene expression plots with sex-specific correlations (Fig. 7). These CpG-gene sets are listed in Additional file 8 along with genomic region.

## Discussion

In this study of the relationship between age and genome-wide DNA methylation patterns in whole blood samples collected from a Bangladeshi population, we observed differentially methylated CpGs with respect to age across the entire genome. More age-associated CpGs were observed among women compared to men, but the presence of association with age was consistent across sexes for most age-associated CpGs and the amount of overlap in top CpG remained relatively consistent regardless of the regression model and confounders included. There was a strong correlation between chronological age and the Horvath methylation age prediction model [40] among both sexes. However, we observed limited overlap between the most significant (*p* < 5 x 10^−8^) age-associated CpGs identified in this work and the CpGs used in the Horvath calculator which is expected as explained in a recent review [51]. Alternative explanations include that there are differences in epigenetic aging features due to tissue type and/or population between our data and the data used to train existing DNA methylation aging models.

We observed similar enrichment in genomic features for age-associated CpGs between sexes. When comparing all CpGs to age-associated CpGs, islands were strongly enriched for age-associated sites, with weaker enrichment for age-associated CpGs in shore regions. Age-associated CpGs were depleted in shelf and open sea regions. We observed enrichment for age-associated CpGs in intergenic regions, with general depletion in gene regions.

Among age-associated CpGs, islands contained sites that were almost exclusively hyper-methylated with increasing age, while shelf and open sea regions contained more hypo- as compared to hyper-methylated sites. Among all age-associated CpGs on the 450 K array, hyper-methylation was approximately twice as common as hypo-methylation, and enrichment for hyper-methylated sites was present in all categories defined according to proximity to gene/TSS. The observation that age-associated hyper-methylation tends to occur in islands and promoter regions [52] while hypo-methylation tends to occur in shelf, shore, and open sea regions is consistent with previous literature [53]. The observed enrichment of age-associated CpGs in island regions (with depletion in open sea and body regions) is also consistent with previous literature [53].

Sex differences in methylation patterns have been observed in studies of both newborns and adults and in different tissue types (e.g., blood and saliva) [54–60]. During preimplantation embryo development, the demethylation process is much faster in males than in females [61], and several prior studies have demonstrated that most age-associated CpG sites showed a higher methylation in females compared to males [9, 32, 62, 63]; however, our results based on our top 100 age-associated CpGs do not support this conclusion (data not shown).

To our knowledge, there are no genome-wide epidemiologic studies that have characterized the association between age and DNA methylation in blood among males and females separately. There are at least 5 studies which specifically investigated sex-specific methylation changes with age. However, these studies, have focused on specific genome locations or were conducted within other tissue types [64–67]. Sex-based differences in the epigenetic aging process could be related to the observation that females and males have different rates of disease incidence for many age-related diseases and different risk thresholds for susceptibility factors to those diseases.

Some of the specific age-associated CpGs identified using blood within the current study are likely to be observed when evaluated in other tissues, however, important considerations include the tissue type and all samples coming from same individuals. A recent paper by Zhu et al. 2018 [68], evaluated age-associated DNA methylation from multiple large publicly available datasets and were able to conduct sub-analyses using methylation across different tissues from the same set of individuals. These authors demonstrated that many age-associated methylation sites are shared across tissue types (as much as 70% or more), however, the pattern is dependent on the specific CpG site and the specific tissues that are being compared [68]. They highlight matching on individual is a key condition when looking at age-related methylation across tissues. Future studies should assess the tissue-independence of our results using methylation data from studies of diverse tissues types obtained from multi-tissue donors, such as the Genotype-Tissue Expression (GTEx) project [69].

The association between increasing methylation in promoter regions and decreasing corresponding gene expression levels has been widely observed in blood, and is believed to reflect epigenetic silencing of promoters [30, 53, 70]. Likewise, a negative association between gene expression and gene body methylation has been demonstrated in blood of various populations [71, 72], but the functional importance of non-promoter region methylation associations with expression are not well understood. Hypothesized mechanisms including modulation of chromatin structure, regulation of alternative promoters, or nucleosome positioning. In an attempt to understand the potential gene-regulatory roles of our top 100 age-associated CpGs within each sex, we examined those CpGs that were assigned to a gene and observed a significant association with expression in 3/417 in the male set and 11/538 in the female set. Thus, the potential regulatory roles for the vast majority of these age-associated CpGs are unclear since they generally are not associated with expression which has been observed with other age-related CpGs [51]. However, these CpGs still tend to occur in promoter regions (TSS1500 or TSS200) (Additional file 4) and a potential difference in age-related variably methylated positions (aVMPs) in males compared to females may explain why we have observed a higher number of CpG sites correlated with gene expression compared with previous studies [53], but, none of our top 100 age-associated CpG sites were contained in that list [73]. Of the 276 CpGs determined to be different based on sex at birth [65] using a *p* of 5 × 10^–8^ (like in our paper), we find that only 2 CpGs are significantly associated with age in our model which adjusted for sex and smoking, but in the same model we observe that methylation for 180 out of these 276 are significantly different based on the sex *p*-value. The regression coefficient for age ranges from − 0.197 to 0.143 (not shown).

Age prediction models using methylation at CpGs (i.e., epigenetic clock or biological aging) have been shown to predict aging-related outcomes, such as all-cause mortality [43], cognitive and physical functions [42], Down syndrome [74], and cancers of the lung, breast, kidney, and blood [75]. These studies demonstrate that a surrogate tissue (blood) is useful for detecting accelerated aging effects that predispose to aging-related diseases of other tissues and that implementation of screening and subsequent early diagnosis could help improve the effectiveness of targeted interventions and prognoses for at-risk populations [51]. There is also the potential for risk assessment in an individual’s family members by investigating key disease-associated methylation markers that demonstrate similar features inter-generationally [76, 77]. However, this research is complex and at a very early stage [78]. Poor correlation has been observed between epigenetic clock predictors (Hannum [38] or Horvath [40] methylation age) and telomere length, however, both have been observed to have significant independent associations with age and mortality [79]. This suggests different pathways/mechanisms are being represented by telomere and DNA methylation markers [79]. Developing methods to combine information from these and other biomarkers of biological aging could provide predictions regarding which patients to target for interventions to improve overall quality of life and survival.

All epigenome-wide associations studies need to consider adjustment for cell type composition. When DNA methylation is assessed in whole blood we need to adjust for leukocyte subtypes, which are known to be heterogeneous with respect to methylation patterns [59, 60]. Different proportions of blood cell types exist between females and males; therefore, addressing cell-type proportions related to sex impacts the number of significant CpGs observed [62, 80]. Therefore, we utilized a statistical method to infer cell type fractions in our samples; the assumptions of the statistical method have been described elsewhere [81, 82]. There were two methods we considered. The first is MethylSectrum [48], which estimates cellular proportions using a reference data set of cell-type specific DNA methylation. The second method, and the primary method used in our work, is a reference-free method [47] that estimates latent variables (including cell type composition factors) using a statistical formula based on an empirical test of the variance explained; hence, this method is not restricted to estimation of only 6 cell-types and can capture additional variables such as experimental batch. In our study, we observed a pattern of asymmetry (much larger number of significant beta values above 0 compared to below 0) while using the MethylSectrum method which was not observed when using the reference-free method. This observation may suggest that the estimates produced by the reference-based MethylSpectrum method, often used in other studies [48, 57] could be affected by unmeasured confounders, and the reference data used may not be ideal for all population world-wide.

There are several reasons we may have observed a larger number of significant age-associated CpGs among females compared to males. There was a larger sample of females compared with males which means there is a power difference between the sex-stratified analyses. The age distribution is more variable (i.e., wider range of ages) among females potentially contributing to the small *p*-values observed among females. There were many more males who were current or former smokers compared with females, thus an additional analysis adjusting for smoking was conducted and is included in the additional files.

Strengths of this study include the relatively large sample size and the availability of genome-wide DNA methylation and expression data from a population-based sample. In addition, very few studies of DNA methylation have been conducted in South Asian individuals. While previous studies have demonstrated associations between age and DNA methylation markers, we were also able to evaluate expression of genes residing near our age-associated CpG sites.

## Conclusions

Our results suggest a similar feature of age-associated CpGs across the genome for males and females. Consistent with prior studies, age-associated CpG sites residing in island and promoter regions tend to be hyper-methylated with increasing age, while age-related CpGs residing in shelf and open sea, regions tend to be hypo-methylated with increasing age. Enrichment of age-associated CpGs occurs in island regions while depletion of age-associated CpGs is observed in open sea, shelf, and gene body regions. Additional studies need to confirm the associations observed in this study and assess potential differences across populations. Future work utilizing multiple epigenetic datasets will likely lead to an enhanced understanding of the role epigenetic factors play in the development of age-associated diseases. In addition, utilizing methylation-based age-prediction models (i.e., biological age) may allow a more accurate categorization of individual disease-specific risks compared with the traditional use of chronological age.

## Additional files

Additional file 1: Age distribution for males and females in BEST. (PDF 55 kb)

Additional file 2: Top 50 results based on *p*-value for analysis methods Methylspectrum and RefFreeEWAS. (PDF 244 kb)

Additional file 3: Number of top 100 age-associated CpGs in common between analysis methdos Methylspectrum and RefFreeEWAS. (PDF 222 kb)

Additional file 4: Number of top 100 age-associated CpGs in common between different RefFreeEWAS models. (PDF 72 kb)

Additional file 5: Age distribution for males and females in HEALS. (PDF 56 kb)

Additional file 6: Top 100 results across BEST (original) and HEALS (validation) datasets based on top age-associated CpGs observed in BEST. (PDF 69 kb)

Additional file 7: Number of top 91 age-associated CpGs in common between different RefFreeEWAS models using original dataset and a) top 100 age-associated CpGs in Validation dataset or b) significant (< 5e-8) age-associated CpGs in the Validation dataset (PDF 69 kb)

Additional file 8: UCSC genome location information for a subset of the top 100 age-associated CpGs observed to be significantly associated with its Illumina assigned gene expression (PDF 112 kb)

## Data Availability

The datasets used and/or analyzed during the current study are available from the corresponding author on reasonable request.

## Abbreviations

aVMPs: Age-related variably methylated positions
BEST: Bangladesh Vitamin E and Selenium Trial
bp: basepairs
CD4T): T-helper
CpG: 5’—C—phosphate—G—3’
DNA: Deoxyribonucleic acid
Gran: Granulocytes
GTEx: Genotype-Tissue Expression
HEALS: Health Effects of Arsenic Longitudinal Study
Mono: Monocytes
NK: Natural killer
r: correlation
RNA: Ribonucleic acid
SD: Standard deviation
SEs: Standard errors
TSS: Transcription start site
UTR: Untranslated region

## References

[1] Lee KWK, Pausova Z (2013). Cigarette smoking and DNA methylation. Front Epigenomics Epigenetics.

[2] Milagro FI, Mansego ML, De Miguel C, Martínez JA (2013). Dietary factors, epigenetic modifications and obesity outcomes: progresses and perspectives. Mol Asp Med.

[3] Li X, Wang Y, Zhang Z, Yao X, Ge J, Zhao Y (2013). Correlation of MLH1 and MGMT methylation levels between peripheral blood leukocytes and colorectal tissue DNA samples in colorectal cancer patients. Oncol Lett.

[4] Ronn T, Volkov P, Gillberg L, Kokosar M, Perfilyev A, Jacobsen AL (2015). Impact of age, BMI and HbA1c levels on the genome-wide DNA methylation and mRNA expression patterns in human adipose tissue and identification of epigenetic biomarkers in blood. Hum Mol Genet.

[5] Van Bemmel D, Lenz P, Liao LM, Baris D, Sternberg LR, Warner A (2012). Correlation of LINE-1 methylation levels in patient-matched buffy coat, serum, buccal cell, and bladder tumor tissue DNA samples. Cancer Epidemiol Biomark Prev.

[6] Walton E, Hass J, Liu J, Roffman JL, Bernardoni F, Roessner V (2016). Correspondence of DNA methylation between blood and brain tissue and its application to schizophrenia research. Schizophr Bull.

[7] Zhang FF, Cardarelli R, Carroll J, Fulda KG, Kaur M, Gonzalez K (2011). Significant differences in global genomic DNA methylation by gender and race/ethnicity in peripheral blood. Epigenetics.

[8] Boks MP, Derks EM, Weisenberger DJ, Strengman E, Janson E, Sommer IE (2009). The relationship of DNA methylation with age, gender and genotype in twins and healthy controls. PLoS One.

[9] Liu J, Morgan M, Hutchison K, Calhoun VD (2010). A study of the influence of sex on genome wide methylation. PLoS One.

[10] Horvath S, Zhang Y, Langfelder P, Kahn RS, Boks MPM, van Eijk K (2012). Aging effects on DNA methylation modules in human brain and blood tissue. Genome Biol.

[11] Bell CG, Xia Y, Yuan W, Gao F, Ward K, Roos L (2016). Novel regional age-associated DNA methylation changes within human common disease-associated loci. Genome Biol.

[12] Rodríguez-Rodero S, Fernández-Morera JL, Fernandez AF, Menéndez-Torre E, Fraga MF (2010). Epigenetic regulation of aging. Discov Med.

[13] Mugatroyd C, Wu Y, Bockmühl Y, Spengler D (2010). The janus face of DNA methylation in aging. Aging (Albany NY).

[14] Teschendorff AE, Menon U, Gentry-Maharaj A, Ramus SJ, Weisenberger DJ, Shen H (2010). Age-dependent DNA methylation of genes that are suppressed in stem cells is a hallmark of cancer. Genome Res.

[15] Bollati V, Schwartz J, Wright R, Litonjua A, Tarantini L, Suh H (2009). Decline in genomic DNA methylation through aging in a cohort of elderly subjects. Mech Ageing Dev.

[16] Christensen BC, Houseman EA, Marsit CJ, Zheng S, Wrensch MR, Wiemels JL (2009). Aging and environmental exposures alter tissue-specific DNA methylation dependent upon CPG island context. PLoS Genet.

[17] Fraga MF, Esteller M (2007). Epigenetics and aging: the targets and the marks. Trends Genet.

[18] Fraga MF, Agrelo R, Esteller M (2007). Cross-talk between aging and cancer: The epigenetic language. Ann N Y Acad Sci.

[19] Banister CE, Koestler DC, Maccani MA, Padbury JF, Andres Houseman E, Marsit CJ (2011). Infant growth restriction is associated with distinct patterns of DNA methylation in human placentas. Epigenetics.

[20] Ehrlich M (2002). DNA methylation in cancer: too much, but also too little. Oncogene.

[21] Toperoff G, Aran D, Kark JD, Rosenberg M, Dubnikov T, Nissan B (2012). Genome-wide survey reveals predisposing diabetes type 2-related DNA methylation variations in human peripheral blood. Hum Mol Genet.

[22] Post W (1999). Methylation of the estrogen receptor gene is associated with aging and atherosclerosis in the cardiovascular system. Cardiovasc Res.

[23] Richardson B (2003). Impact of aging on DNA methylation. Ageing Res Rev.

[24] Fraga M, Ballestar E, Paz M, Ropero S, Setien F (2004). From the cover: epigenetic differences arise during the lifetime of monozygotic twins. Proc Natl Acad Sci.

[25] Ribel-Madsen R, Fraga MF, Jacobsen S, Bork-Jensen J, Lara E, Calvanese V (2012). Genome-wide analysis of DNA methylation differences in muscle and fat from monozygotic twins discordant for type 2 diabetes. PLoS One.

[26] Kaminsky ZA, Tang T, Wang S-C, Ptak C, Oh GHT, Wong AHC (2009). DNA methylation profiles in monozygotic and dizygotic twins. Nat Genet.

[27] Nilsson E, Jansson PA, Perfilyev A, Volkov P, Pedersen M, Svensson MK (2014). Altered DNA methylation and differential expression of genes influencing metabolism and inflammation in adipose tissue from subjects with type 2 diabetes. Diabetes.

[28] Brøns C, Jensen CB, Storgaard H, Alibegovic A, Jacobsen S, Nilsson E (2008). Mitochondrial function in skeletal muscle is normal and unrelated to insulin action in young men born with low birth weight. J Clin Endocrinol Metab.

[29] Barker DJP (1997). Maternal nutrition, fetal nutrition, and disease in later life. Nutrition..

[30] Xu Z, Taylor JA (2014). Genome-wide age-related DNA methylation changes in blood and other tissues relate to histone modification, expression and cancer. Carcinogenesis.

[31] Jones PA (2012). Functions of DNA methylation: islands, start sites, gene bodies and beyond. Nat Rev Genet.

[32] Numata S, Ye T, Hyde TM, Guitart-Navarro X, Tao R, Wininger M (2012). DNA methylation signatures in development and aging of the human prefrontal cortex. Am J Hum Genet.

[33] Koch CM, Wagner W (2011). Epigenetic-aging-signature to determine age in different tissues. Aging (Albany NY).

[34] Hernandez DG, Nalls MA, Gibbs JR, Arepalli S, van der Brug M, Chong S (2011). Distinct DNA methylation changes highly correlated with chronological age in the human brain. Hum Mol Genet.

[35] Rakyan VK, Down TA, Maslau S, Andrew T, Yang TP, Beyan H (2010). Human aging-associated DNA hypermethylation occurs preferentially at bivalent chromatin domains. Genome Res.

[36] Florath I, Butterbach K, Müller H, Bewerunge-hudler M, Brenner H (2014). Cross-sectional and longitudinal changes in DNA methylation with age: an epigenome-wide analysis revealing over 60 novel age-associated CpG sites. Hum Mol Genet.

[37] Johansson Å, Enroth S, Gyllensten U (2013). Continuous aging of the human DNA Methylome throughout the human lifespan. PLoS One.

[38] Hannum G, Guinney J, Zhao L, Zhang L, Hughes G, Sadda S (2013). Genome-wide methylation profiles reveal quantitative views of human aging rates. Mol Cell.

[39] Bocklandt S, Lin W, Sehl ME, Sánchez FJ, Sinsheimer JS, Horvath S (2011). Epigenetic predictor of age. PLoS One.

[40] Horvath S (2013). DNA methylation age of human tissues and cell types DNA methylation age of human tissues and cell types.

[41] Ling C, Groop L (2009). Epigenetics: A molecular link between environmental factors and type 2 diabetes. Diabetes.

[42] Marioni RE, Shah S, McRae AF, Ritchie SJ, Muniz-Terrera G, Harris SE (2015). The epigenetic clock is correlated with physical and cognitive fitness in the Lothian birth cohort 1936. Int J Epidemiol.

[43] Marioni RE, Shah S, McRae AF, Chen BH, Colicino E, Harris SE (2015). DNA methylation age of blood predicts all-cause mortality in later life. Genome Biol.

[44] Sandoval J, Heyn HA, Moran S, Serra-Musach J, Pujana MA, Bibikova M (2011). Validation of a DNA methylation microarray for 450,000 CpG sites in the human genome. Epigenetics.

[45] Argos M, Rahman M, Parvez F, Dignam J, Islam T, Quasem I (2013). Baseline comorbidities in a skin cancer prevention trial in Bangladesh. Eur J Clin Investig.

[46] Argos M, Chen L, Jasmine F, Tong L, Pierce BL, Roy S (2015). Gene-specific differential DNA methylation and chronic arsenic exposure in an epigenome-wide association study of adults in Bangladesh. Environ Health Perspect.

[47] Houseman EA, Molitor J, Marsit CJ (2014). Reference-free cell mixture adjustments in analysis of DNA methylation data. Bioinformatics.

[48] Houseman EA, Accomando WP, Koestler DC, Christensen BC, Marsit CJ, Nelson HH (2012). DNA methylation arrays as surrogate measures of cell mixture distribution. BMC Bioinformatics.

[49] R Core Team (2016). R: A Language and Environment for Statistical Computing.

[50] Ahsan H, Chen Y, Parvez F, Argos M, Hussain AI, Momotaj H (2006). Health effects of arsenic longitudinal study (HEALS): description of a multidisciplinary epidemiologic investigation. J Expo Sci Environ Epidemiol.

[51] Horvath Steve, Raj Kenneth (2018). DNA methylation-based biomarkers and the epigenetic clock theory of ageing. Nature Reviews Genetics.

[52] Zampieri M, Ciccarone F, Calabrese R, Franceschi C, Bürkle A, Caiafa P (2015). Reconfiguration of DNA methylation in aging. Mech Ageing Dev.

[53] Steegenga WT, Boekschoten MV, Lute C, Hooiveld GJ, De Groot PJ, Morris TJ (2014). Genome-wide age-related changes in DNA methylation and gene expression in human PBMCs. Age (Omaha).

[54] Kinoshita M, Numata S, Tajima A, Ohi K, Hashimoto R, Shimodera S (2014). Aberrant DNA methylation of blood in schizophrenia by adjusting for estimated cellular proportions. NeuroMolecular Med.

[55] Liu Y, Aryee MJ, Padyukov L, Fallin MD, Hesselberg E, Runarsson A (2013). Epigenome-wide association data implicate DNA methylation as an intermediary of genetic risk in rheumatoid arthritis. Nat Biotechnol.

[56] Lam LL, Emberly E, Fraser HB, Neumann SM, Chen E, Miller GE (2012). Factors underlying variable DNA methylation in a human community cohort. Proc Natl Acad Sci.

[57] Jaffe AE, Irizarry RA (2014). Accounting for cellular heterogeneity is critical in epigenome-wide association studies. Genome Biol.

[58] Guintivano J, Aryee MJ, Kaminsky ZA (2013). A cell epigenotype specific model for the correction of brain cellular heterogeneity bias and its application to age, brain region and major depression. Epigenetics.

[59] Adalsteinsson BT, Gudnason H, Aspelund T, Harris TB, Launer LJ, Eiriksdottir G (2012). Heterogeneity in white blood cells has potential to confound DNA methylation measurements. PLoS One.

[60] Reinius LE, Acevedo N, Joerink M, Pershagen G, Dahlén S-E, Greco D (2012). Differential DNA methylation in purified human blood cells: implications for cell lineage and studies on disease susceptibility. PLoS One.

[61] Guo H, Zhu P, Yan L, Li R, Hu B, Lian Y (2014). The DNA methylation landscape of human early embryos. Nature.

[62] Sun L, Lin J, Du H, Hu C, Huang Z, Lv Z (2014). Gender-specific DNA methylome analysis of a Han Chinese longevity population. Biomed Res Int.

[63] Xu H, Wang F, Liu Y, Yu Y, Gelernter J, Zhang H (2014). Sex-biased methylome and transcriptome in human prefrontal cortex. Hum Mol Genet.

[64] Masser DR, Hadad N, Hunter L, Mangold CA, Unnikrishnan A, Ford MM (2017). Sexually divergent DNA methylation patterns with hippocampal aging. Aging Cell.

[65] Yousefi P, Huen K, Davé V, Barcellos L, Eskenazi B, Holland N (2015). Sex differences in DNA methylation assessed by 450 K BeadChip in newborns. BMC Genomics.

[66] Van DJ, Nivard MG, Willemsen G, Hottenga J, Helmer Q, Dolan CV (2016). Genetic and environmental influences interact with age and sex in shaping the human methylome. Nat Commun.

[67] Naumova AK, Al Tuwaijri A, Morin A, Vaillancout VT, Madore A-M, Berlivet S (2013). Sex- and age-dependent DNA methylation at the 17q12-q21 locus associated with childhood asthma. Hum Genet.

[68] Zhu T, Zheng SC, Paul DS, Horvath S, Teschendorff AE (2018). Cell and tissue type independent age-associated DNA methylation changes are not rare but common. Aging (Albany NY).

[69] GTEx Consortium TGte (2013). The Genotype-Tissue Expression (GTEx) project. Nat Genet.

[70] Mansego M, Milagro F, Zulet M, Moreno-Aliaga M, Martínez J (2015). Differential DNA methylation in relation to age and health risks of obesity. Int J Mol Sci.

[71] Almén MS, Nilsson EK, Jacobsson JA, Kalnina I, Klovins J, Fredriksson R (2014). Genome-wide analysis reveals DNA methylation markers that vary with both age and obesity. Gene.

[72] Habano W, Kawamura K, Iizuka N, Terashima J, Sugai T, Ozawa S (2015). Analysis of DNA methylation landscape reveals the roles of DNA methylation in the regulation of drug metabolizing enzymes. Clin Epigenetics.

[73] Slieker RC, van Iterson M, Luijk R, Beekman M, Zhernakova DV, Moed MH (2016). Age-related accrual of methylomic variability is linked to fundamental ageing mechanisms. Genome Biol.

[74] Horvath S, Garagnani P, Bacalini MG, Pirazzini C, Salvioli S, Gentilini D (2015). Accelerated epigenetic aging in Down syndrome. Aging Cell.

[75] Dugué P-A, Bassett JK, Joo JE, Jung C-H, Ming Wong E, Moreno-Betancur M (2017). DNA methylation-based biological aging and cancer risk and survival: pooled analysis of seven prospective studies. Int J Cancer.

[76] Evert J, Lawler E, Bogan H, Perls T (2003). Morbidity profiles of centenarians: survivors, delayers, and escapers. J Gerontol Med Sci.

[77] Xiao F-H, He Y-H, Li Q-G, Wu H, Luo L-H, Kong Q-P (2015). A genome-wide scan reveals important roles of DNA methylation in human longevity by regulating age-related disease genes. PLoS One.

[78] van Dongen J, Nivard MG, Willemsen G, Hottenga J-J, Helmer Q, Dolan CV (2016). Genetic and environmental influences interact with age and sex in shaping the human methylome. Nat Commun.

[79] Marioni Riccardo E, Harris Sarah E, Shah Sonia, McRae Allan F, von Zglinicki Thomas, Martin-Ruiz Carmen, Wray Naomi R, Visscher Peter M, Deary Ian J (2016). The epigenetic clock and telomere length are independently associated with chronological age and mortality. International Journal of Epidemiology.

[80] Inoshita M, Numata S, Tajima A, Kinoshita M, Umehara H, Yamamori H (2015). Sex differences of leukocytes DNA methylation adjusted for estimated cellular proportions. Biol Sex Differ.

[81] Koestler DC, Avissar-Whiting M, Andres Houseman E, Karagas MR, Marsit CJ (2013). Differential DNA methylation in umbilical cord blood of infants exposed to low levels of arsenic in utero. Environ Health Perspect.

[82] Koestler DC, Christensen BC, Karagas MR, Marsit CJ, Langevin SM, Kelsey KT (2013). Blood-based profiles of DNA methylation predict the underlying distribution of cell types: a validation analysis. Epigenetics.

