# Supplementary figures and images for: The effect of age on DNA methylation in whole blood among Bangladeshi men and women
Source: BMC Genomics. 2019 Sep 10;20:704. doi: 10.1186/s12864-019-6039-9 (PMC6734473; doi:10.1186/s12864-019-6039-9)

**Additional File 1.** Age distribution for males and females in BEST

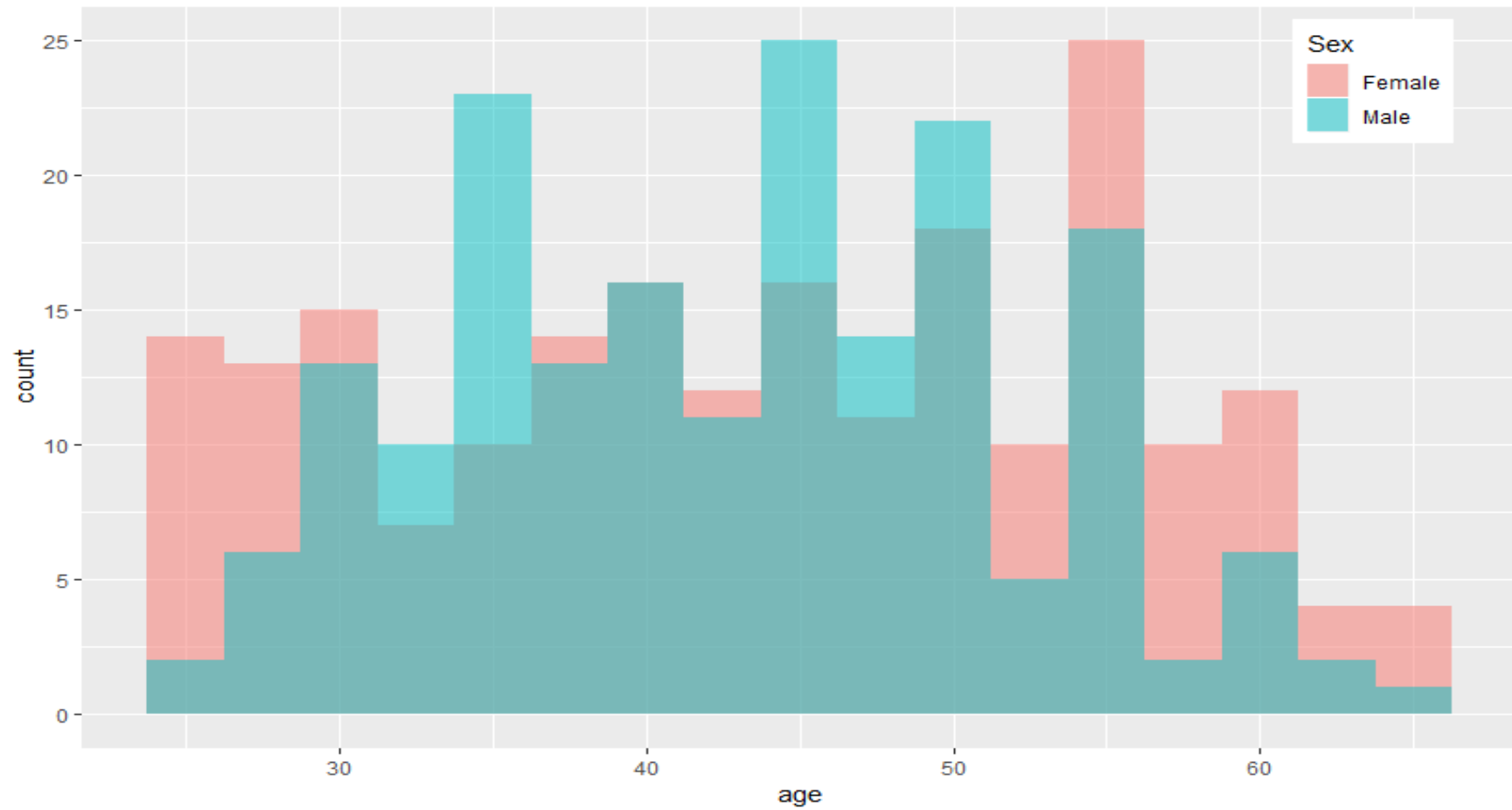

Supplement: Supplementary file 1 — Age distribution for males and females in BEST. (PDF 55 kb) [file 12864_2019_6039_MOESM1_ESM.pdf]

**Additional File 5.** Age distribution for males and females in HEALS

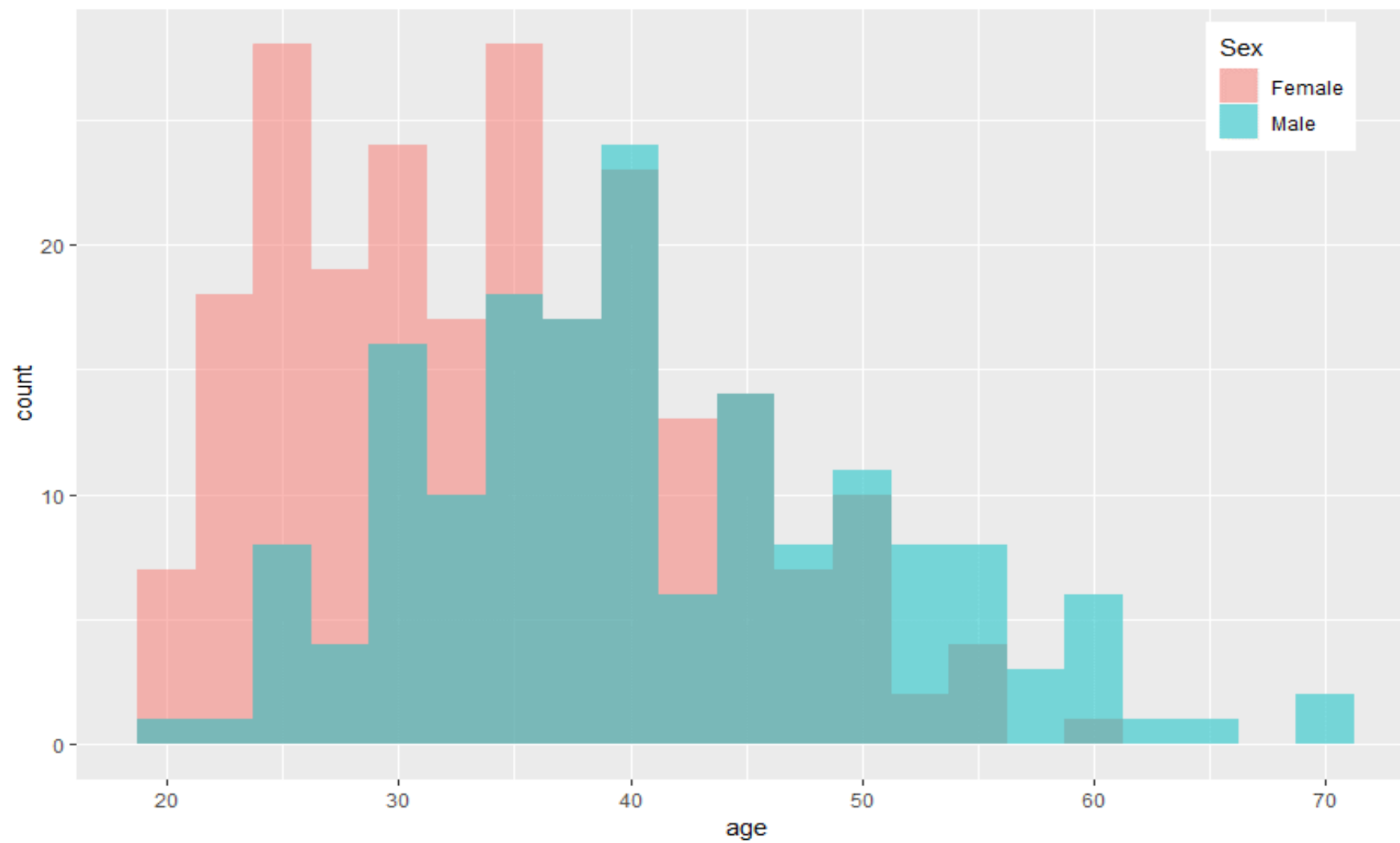

Supplement: Supplementary file 5 — Age distribution for males and females in HEALS. (PDF 56 kb) [file 12864_2019_6039_MOESM5_ESM.pdf]
