# Supplementary material for: The effect of age on DNA methylation in whole blood among Bangladeshi men and women
Source: BMC Genomics. 2019 Sep 10;20:704. doi: 10.1186/s12864-019-6039-9 (PMC6734473; doi:10.1186/s12864-019-6039-9)
Supplement: Supplementary file 2 — Top 50 results based on p-value for analysis methods Methylspectrum and RefFreeEWAS. (PDF 244 kb) [file 12864_2019_6039_MOESM2_ESM.pdf]

**Additional File 2**. Top 50 results based on p-value for analysis methods Methylspectrum and RefFreeEWAS

| Top 50 Methylspectrum Results | | | | Top 50 RefFreeEWAS Results | | | |
|---|---|---|---|---|---|---|---|
| Women | | Men | | Women | | Men | |
| CPG | pvalue | CPG | pvalue | CPG | pvalue | CPG | pvalue |
| cg16867657 | 7.97E-60 | cg16867657 | 4.99E-30 | cg16867657 | 1.49E-71 | cg0032961 | 5.20E-38 |
| cg07553761 | 7.80E-42 | cg07547549 | 4.91E-29 | cg26685941 | 4.91E-48 | cg1686765 | 1.91E-30 |
| cg10501210 | 3.73E-40 | cg06639320 | 1.36E-25 | cg14361627 | 1.12E-47 | cg2157272 | 4.28E-24 |
| cg14361627 | 4.05E-40 | cg10501210 | 2.58E-24 | cg19283806 | 1.48E-46 | cg0754754 | 6.22E-24 |
| cg08097417 | 6.70E-39 | cg08097417 | 1.21E-23 | cg10501210 | 3.27E-46 | cg0487512 | 1.98E-23 |
| cg22454769 | 1.03E-37 | cg14361627 | 6.41E-23 | cg12757011 | 1.99E-45 | cg1302984 | 3.83E-23 |
| cg06639320 | 1.45E-36 | cg21572722 | 1.19E-21 | cg07553761 | 4.87E-42 | cg0708226 | 3.90E-23 |
| cg21572722 | 3.43E-35 | cg07553761 | 1.50E-21 | cg07082267 | 1.33E-41 | cg2245476 | 1.77E-22 |
| cg24724428 | 1.46E-34 | cg10804656 | 2.71E-21 | cg22454769 | 2.43E-40 | cg2407970 | 4.11E-22 |
| cg10804656 | 1.85E-34 | cg22736354 | 1.96E-20 | cg16181396 | 5.47E-40 | cg1080465 | 4.66E-22 |
| cg00481951 | 7.98E-34 | cg22454769 | 2.13E-20 | cg24079702 | 6.81E-40 | cg0708037 | 6.64E-22 |
| cg17885226 | 9.63E-33 | cg13327545 | 8.84E-19 | cg00481951 | 1.24E-39 | cg2120065 | 8.21E-22 |
| cg04875128 | 7.19E-32 | cg13954457 | 1.11E-18 | cg07408456 | 1.81E-35 | cg1853055 | 1.66E-21 |
| cg12757011 | 1.50E-30 | cg04875128 | 3.28E-18 | cg17280346 | 1.33E-34 | cg1436162 | 2.58E-21 |
| cg23500537 | 3.85E-30 | cg25478614 | 6.77E-18 | cg08097417 | 1.36E-34 | cg2412582 | 4.52E-20 |
| cg17110586 | 6.75E-30 | cg11970349 | 7.38E-18 | cg01763090 | 1.69E-34 | cg1050121 | 4.63E-20 |
| cg07955995 | 9.55E-30 | cg07955995 | 1.36E-17 | cg16312514 | 2.75E-34 | cg1169370 | 1.10E-19 |
| cg01844642 | 1.54E-29 | cg24724428 | 2.44E-17 | cg06493994 | 9.45E-34 | cg0355522 | 1.57E-19 |
| cg09401099 | 1.64E-29 | cg03771840 | 4.31E-17 | cg23744638 | 1.61E-33 | cg0663932 | 2.01E-19 |
| cg18473521 | 3.05E-29 | cg03473532 | 4.40E-17 | cg10137837 | 4.31E-33 | cg0215352 | 2.37E-19 |
| cg07082267 | 9.45E-29 | cg23174607 | 7.37E-17 | cg06784991 | 1.15E-32 | cg1616679 | 2.41E-19 |
| cg06493994 | 9.69E-29 | cg17110586 | 7.61E-17 | cg25410668 | 1.28E-32 | cg2462729 | 2.50E-19 |
| cg18064714 | 9.82E-29 | cg01153166 | 9.40E-17 | cg26161329 | 2.01E-32 | cg0034309 | 2.56E-19 |
| cg24079702 | 1.03E-28 | cg07080372 | 1.05E-16 | cg11220950 | 8.01E-32 | cg0755376 | 2.83E-19 |
| cg14692377 | 1.65E-28 | cg14472366 | 1.30E-16 | cg24430580 | 9.95E-32 | cg0162016 | 9.44E-19 |
| cg25410668 | 1.77E-28 | cg20273670 | 2.17E-16 | cg24724428 | 1.82E-31 | cg0265026 | 1.23E-18 |
| cg07547549 | 2.52E-28 | cg23500537 | 3.03E-16 | cg19344626 | 1.85E-31 | cg1641923 | 2.01E-18 |
| cg06782035 | 3.27E-28 | cg09499629 | 3.68E-16 | cg04266460 | 2.14E-30 | cg1014953 | 6.00E-18 |
| cg05991454 | 6.95E-28 | cg23078123 | 4.26E-16 | cg17110586 | 3.45E-30 | cg0314912 | 6.40E-18 |
| cg08160331 | 8.18E-28 | cg19283806 | 4.36E-16 | cg14674720 | 4.76E-30 | cg0320016 | 1.59E-17 |
| cg12934382 | 1.38E-26 | cg15341124 | 6.28E-16 | cg10778288 | 8.01E-30 | cg1395445 | 1.80E-17 |
| cg22682811 | 3.29E-26 | cg04400972 | 1.14E-15 | cg17760405 | 9.56E-30 | cg0750238 | 1.85E-17 |
| cg00503840 | 3.76E-26 | cg05024939 | 1.20E-15 | cg20294304 | 1.25E-29 | cg0244722 | 2.13E-17 |
| cg06784991 | 4.51E-26 | cg09401099 | 1.30E-15 | cg04400972 | 1.51E-29 | cg0980967 | 4.51E-17 |
| cg06279276 | 6.07E-26 | cg07082267 | 1.56E-15 | cg04875128 | 2.32E-29 | cg2525672 | 5.34E-17 |
| cg00664406 | 7.11E-26 | cg02650266 | 2.04E-15 | cg19863655 | 2.52E-29 | cg0842673 | 7.84E-17 |
| cg26290632 | 7.57E-26 | cg21200656 | 2.10E-15 | cg05308819 | 3.45E-29 | cg2005276 | 7.87E-17 |
| cg16419235 | 1.05E-25 | cg12934382 | 2.37E-15 | cg21572722 | 4.84E-29 | cg2489206 | 1.04E-16 |
| cg13327545 | 1.32E-25 | cg05991454 | 2.85E-15 | cg25334393 | 5.43E-29 | cg1781690 | 1.39E-16 |
| cg20426994 | 1.38E-25 | cg03555227 | 3.15E-15 | cg11084334 | 7.61E-29 | cg1001053 | 2.10E-16 |
| cg11220950 | 1.65E-25 | cg17885226 | 3.26E-15 | cg03646916 | 8.90E-29 | cg1972284 | 2.22E-16 |
| cg16181396 | 2.02E-25 | cg27252696 | 3.74E-15 | cg15341124 | 1.01E-28 | cg2279670 | 2.39E-16 |

| | | | | | | | |
|---|---|---|---|---|---|---|---|
| cg15618978 | 3.58E-25 | cg16419235 | 4.01E-15 | cg06782035 | 1.01E-28 | cg1332754 | 2.40E-16 |
| cg26161329 | 4.71E-25 | cg04090392 | 4.32E-15 | cg01620164 | 1.01E-28 | cg1802663 | 3.12E-16 |
| cg01763090 | 7.83E-25 | cg06493994 | 4.84E-15 | cg20818778 | 2.06E-28 | cg0494057 | 3.46E-16 |
| cg10242160 | 7.99E-25 | cg09578475 | 5.67E-15 | cg10149533 | 2.67E-28 | cg0521389 | 3.99E-16 |
| cg04400972 | 9.53E-25 | cg05835105 | 5.75E-15 | cg19784428 | 2.71E-28 | cg1117699 | 4.52E-16 |
| cg15557036 | 9.64E-25 | cg18473521 | 6.21E-15 | cg12317815 | 3.90E-28 | cg2262009 | 4.76E-16 |
| cg11436113 | 1.07E-24 | cg26161329 | 6.53E-15 | cg03530962 | 4.18E-28 | cg2187421 | 6.85E-16 |
| cg02650266 | 1.68E-24 | cg21159778 | 6.91E-15 | cg02391713 | 5.19E-28 | cg0347353 | 1.24E-15 |
