# Supplementary material for: The effect of age on DNA methylation in whole blood among Bangladeshi men and women
Source: BMC Genomics. 2019 Sep 10;20:704. doi: 10.1186/s12864-019-6039-9 (PMC6734473; doi:10.1186/s12864-019-6039-9)
Supplement: Supplementary file 3 — Number of top 100 age-associated CpGs in common between analysis methdos Methylspectrum and RefFreeEWAS. (PDF 222 kb) [file 12864_2019_6039_MOESM3_ESM.pdf]

Additional File 3. Number of top 100 age-associated CpGs in common between analysis methdos Methylspectrum and RefFreeEWAS

	meth_all	meth_men	meth_women	Ref_all	Ref_men	Ref_women	Horvath Clock CpGs
meth_all	100	60	81	0	36	51	3
meth_men		100	47	0	38	37	3
meth_women			100	0	28	51	2
Ref_all				100	0	0	0
Ref_men					100	32	4
Ref_women						100	2
Horvath Clock CpGs							100
