# Supplementary material for: The effect of age on DNA methylation in whole blood among Bangladeshi men and women
Source: BMC Genomics. 2019 Sep 10;20:704. doi: 10.1186/s12864-019-6039-9 (PMC6734473; doi:10.1186/s12864-019-6039-9)
Supplement: Supplementary file 4 — Number of top 100 age-associated CpGs in common between different RefFreeEWAS models. (PDF 72 kb) [file 12864_2019_6039_MOESM4_ESM.pdf]

Additional File Table 4. Number of top 100 age-associated CpGs in common between different RefFreeEWAS models

	Ref_all*	Ref_all_int'	Ref_men#	Ref_women#	Ref_all_sm^	Ref_men_sm~	Ref_women_sm~	Horvath Clock CpGs
Ref_all*	100	25	34	32	43	36	21	2
Ref_all_int`		100	19	27	31	18	21	2
Ref_men#			100	31	25	40	47	3
Ref_women#				100	43	28	39	3
Ref_all_sm^					100	34	43	2
Ref_men_sm~						100	28	5
Ref_women_sm~							100	5
Horvath Clock CpGs								100

* Model factors include sex and age

` Model factors include sex, age, and sex*age

Model factors include age

^ Model factors include age, sex, and smoking

~ Model factors include age and smoking
