# Supplementary material for: The effect of age on DNA methylation in whole blood among Bangladeshi men and women
Source: BMC Genomics. 2019 Sep 10;20:704. doi: 10.1186/s12864-019-6039-9 (PMC6734473; doi:10.1186/s12864-019-6039-9)
Supplement: Supplementary file 6 — Top 100 results across BEST (original) and HEALS (validation) datasets based on top age-associated CpGs observed in BEST. (PDF 69 kb) [file 12864_2019_6039_MOESM6_ESM.pdf]

Additional File Table 6. Top 100 results across BEST (original) and HEALS (validation) datasets based on top age-associated CpGs observed in BEST

			Men		
	Original		validation		
CPG.Labels	beta	beta_p	age_beta	age_p	beta ratio (orginal/validation)
cg00329615	-0.003889522	5.20E-38	-0.00404076	1.89E-10	0.962571946
cg16867657	0.004175617	1.91E-30	0.00519592	2.76E-49	0.803633828
cg21572722	0.001748031	4.28E-24	0.002177773	6.18E-57	0.802669002
cg07547549	0.002312491	6.22E-24	0.002228514	1.75E-26	1.037682905
cg04875128	0.004575238	1.98E-23	0.004741795	2.28E-24	0.964874767
cg13029847	0.00050631	3.83E-23	0.000599544	9.74E-09	0.844490955
cg07082267	-0.001921664	3.90E-23	-0.002087985	1.65E-27	0.920343622
cg22454769	0.003396999	1.77E-22	0.005235923	2.13E-39	0.648787033
cg24079702	0.002653708	4.11E-22	NA	NA	#VALUE!
cg10804656	0.002584895	4.66E-22	0.003107597	3.69E-13	0.83179855
cg07080372	-0.001510129	6.64E-22	-0.001378917	2.20E-22	1.095155499
cg21200656	0.001209527	8.21E-22	0.001187083	3.25E-13	1.018907018
cg18530551	0.000863614	1.66E-21	0.000421471	0.00028	2.049048344
cg14361627	0.002142517	2.58E-21	NA	NA	#VALUE!
cg24125828	0.002559125	4.52E-20	0.003318505	2.52E-22	0.771168052
cg10501210	-0.006143167	4.63E-20	-0.005842812	3.38E-36	1.051405824
cg11693709	-0.002757297	1.10E-19	-0.002493463	2.66E-10	1.105810321
cg03555227	0.001014472	1.57E-19	0.001149691	4.88E-10	0.882386516
cg06639320	0.002394802	2.01E-19	0.003909853	2.09E-38	0.612504401
cg02153528	-0.000789892	2.37E-19	-0.000973744	5.20E-21	0.811191194
cg16166796	0.000550088	2.41E-19	0.000591041	7.16E-05	0.930711332
cg24627299	-0.001826906	2.50E-19	-0.001501294	3.14E-06	1.216887636
cg00343092	-0.001466593	2.56E-19	-0.000922729	1.67E-06	1.589407446
cg07553761	0.002769141	2.83E-19	0.004710469	3.72E-24	0.58786952
cg01620164	-0.007188427	9.44E-19	-0.009732436	3.76E-16	0.738605089
cg02650266	0.001304526	1.23E-18	0.001555106	1.14E-08	0.838866578
cg16419235	0.001344601	2.01E-18	0.001850532	2.35E-11	0.726602652
cg10149533	-0.002057843	6.00E-18	NA	NA	#VALUE!
cg03149128	0.001992724	6.40E-18	0.002045558	4.06E-13	0.974171261
cg03200166	0.001371903	1.59E-17	0.001028424	6.11E-06	1.333985727
cg13954457	0.001903465	1.80E-17	0.001809919	3.50E-07	1.051684756
cg07502389	0.00105965	1.85E-17	0.001544254	2.46E-17	0.68618923
cg02447229	0.000720962	2.13E-17	0.00049958	0.00026	1.443136321
cg09809672	-0.002795356	4.51E-17	-0.002556365	2.51E-06	1.093488513
cg25256723	-0.001590412	5.34E-17	-0.001533304	3.12E-18	1.037245483
cg08426733	-0.000938279	7.84E-17	-0.000868145	1.01E-14	1.080786281
cg20052760	-0.002025233	7.87E-17	-0.002134225	6.89E-15	0.948931454
cg24892069	-0.003566066	1.04E-16	-0.00452439	5.04E-12	0.788187285
cg17816908	0.000767015	1.39E-16	NA	NA	#VALUE!
cg10010533	0.001166007	2.10E-16	0.001522934	7.20E-05	0.765632275
cg19722847	-0.001195731	2.22E-16	-0.000858212	1.19E-07	1.39328252

cg22796704	-0.002812787	2.39E-16	-0.00299745	2.51E-18	0.93839304
cg13327545	0.001659135	2.40E-16	0.001963005	4.73E-20	0.845201447
cg18026631	0.001063364	3.12E-16	0.000473162	0.00011	2.247355142
cg04940570	0.001432174	3.46E-16	0.001791522	1.53E-06	0.799417297
cg05213896	0.003030432	3.99E-16	0.003309315	1.57E-21	0.915727848
cg11176990	0.000665883	4.52E-16	0.000846991	5.10E-22	0.786174628
cg22620090	0.00132427	4.76E-16	0.000397552	0.00083	3.331063206
cg21874213	-0.001134905	6.85E-16	-0.001189582	6.98E-13	0.954036308
cg03473532	-0.002077605	1.24E-15	-0.002422296	4.91E-14	0.857700781
cg24724428	0.002414545	1.26E-15	0.002861908	3.81E-21	0.843683634
cg19935040	0.003438105	1.42E-15	0.003664568	2.72E-17	0.938202091
cg00046625	0.000533126	1.42E-15	0.0005624	0.02662	0.94794869
cg14757228	0.001902497	1.68E-15	0.000965275	3.37E-07	1.970937595
cg24466241	0.001944477	2.08E-15	0.002183951	1.99E-08	0.890348347
cg15618978	0.002394802	2.11E-15	NA	NA	#VALUE!
cg23078123	-0.001887183	2.13E-15	-0.002193527	7.30E-18	0.860342058
cg07806886	0.000824957	2.24E-15	0.001050542	2.60E-08	0.785267688
cg26330518	0.000612902	2.30E-15	0.000550235	9.58E-11	1.113891625
cg12882697	0.000642113	2.37E-15	0.000582893	0.00464	1.101596133
cg17885226	0.001239241	3.37E-15	0.00182113	1.86E-09	0.680479395
cg03172991	-0.001110808	4.66E-15	-0.000906806	2.40E-08	1.224967106
cg15277914	-0.002219193	5.20E-15	-0.002028101	2.72E-06	1.094222147
cg23500537	0.001801054	5.21E-15	0.002464404	2.36E-27	0.730827131
cg15222899	0.000996248	5.30E-15	0.001425546	5.16E-11	0.698853434
cg06879746	-0.001811449	5.48E-15	-0.002308805	2.10E-17	0.784583089
cg22897615	0.002208961	6.65E-15	0.002128207	5.74E-11	1.03794497
cg14979593	-0.001106355	7.08E-15	-0.000895387	3.02E-06	1.235615694
cg03431918	-0.001263115	7.25E-15	-0.001132292	2.47E-17	1.115538814
cg13472192	0.001197778	7.90E-15	0.001453155	6.06E-05	0.824260152
cg23174607	0.001139877	8.16E-15	0.001432901	1.15E-17	0.795503314
cg18473521	0.002614597	8.57E-15	NA	NA	#VALUE!
cg08097417	0.001572154	8.77E-15	0.001864467	1.23E-22	0.843218819
cg06708720	-0.001075121	1.07E-14	-0.001444722	1.23E-23	0.744171676
cg09401099	0.001577684	1.10E-14	0.002774919	3.06E-27	0.568551277
cg11649376	-0.001539249	1.13E-14	-0.002290787	1.24E-24	0.671930211
cg07850154	-0.002320786	1.38E-14	-0.002267791	1.11E-15	1.023368642
cg02900766	0.000893151	1.43E-14	0.001126034	1.94E-12	0.793182803
cg05406635	0.001249836	1.51E-14	0.000975756	0.00308	1.280890217
cg10290276	0.000960643	1.62E-14	0.001280573	4.30E-07	0.750166638
cg26161329	0.001367739	1.66E-14	0.001460683	2.11E-08	0.936369995
cg00573770	-0.002673538	1.73E-14	-0.004184884	2.77E-16	0.638855893
cg23766254	0.00355536	1.92E-14	0.003397958	3.96E-08	1.046322614
cg20273670	0.002593137	2.10E-14	NA	NA	#VALUE!
cg08262002	-0.001790739	2.51E-14	-0.002368363	1.36E-12	0.756108253
cg06900164	0.001688845	2.83E-14	NA	NA	#VALUE!
cg00884093	0.001038202	2.93E-14	0.001765664	9.33E-05	0.587995056
cg17621438	-0.002063905	2.97E-14	-0.002233283	8.46E-16	0.924157131

cg01153166	0.000736672	3.42E-14	0.000919172	5.61E-11	0.801451911
cg03354992	0.001442555	3.63E-14	0.001565807	4.51E-10	0.921285354
cg19283806	-0.003404952	3.69E-14	-0.003666474	1.58E-42	0.928672214
cg19560758	0.000786572	4.01E-14	0.001298945	2.66E-11	0.605546681
cg14692377	0.001503635	4.26E-14	0.001666965	1.94E-10	0.902019508
cg17430979	-0.002561356	4.45E-14	-0.00220592	1.17E-08	1.161128406
cg00309133	-0.000895361	4.47E-14	-0.000722615	4.05E-06	1.23905726
cg12934382	0.004227595	4.55E-14	0.005015185	7.64E-16	0.842958904
cg25371036	-0.001528694	4.62E-14	-0.001457243	1.87E-13	1.049031791
cg20438306	0.001033176	5.20E-14	NA	NA	#VALUE!
cg16932827	-0.001846822	5.86E-14	-0.001569547	1.65E-14	1.176659413
cg01282174	-0.002217035	6.25E-14	-0.002043915	8.07E-13	1.084700531

	Original		Women validation		
CPG.Labels	beta	beta_p	age_beta	age_p	beta ratio (orginal/validation)
cg16867657	0.004344869	1.49E-71	0.005746044	1.24E-56	0.756149669
cg26685941	-0.002557017	4.91E-48	-0.002359223	9.54E-19	1.083838276
cg14361627	0.002373758	1.12E-47	NA	NA	#VALUE!
cg19283806	-0.003513585	1.48E-46	-0.003288239	5.63E-21	1.068531098
cg10501210	-0.007049714	3.27E-46	-0.005694818	8.47E-35	1.23791742
cg12757011	0.001269443	1.99E-45	0.0017729	3.36E-37	0.7160259
cg07553761	0.002858581	4.87E-42	0.003773494	1.60E-16	0.757542292
cg07082267	-0.002169028	1.33E-41	-0.00207837	7.32E-29	1.043619365
cg22454769	0.004193351	2.43E-40	0.004278266	1.93E-31	0.980151949
cg16181396	0.001072671	5.47E-40	0.001638342	2.07E-27	0.654729534
cg24079702	0.00305635	6.81E-40	NA	NA	#VALUE!
cg00481951	0.001513041	1.24E-39	0.001683153	2.82E-19	0.89893283
cg07408456	-0.001483237	1.81E-35	-0.001313053	9.06E-17	1.129609592
cg17280346	0.00275537	1.33E-34	0.00159837	0.00016	1.723862279
cg08097417	0.001698813	1.36E-34	0.001521508	1.34E-19	1.11653198
cg01763090	0.001086387	1.69E-34	0.000924908	4.77E-19	1.174588841
cg16312514	-0.001501899	2.75E-34	-0.001594799	4.19E-08	0.941748033
cg06493994	0.001058359	9.45E-34	0.001397514	1.27E-15	0.757315615
cg23744638	-0.002787714	1.61E-33	-0.001719393	1.02E-05	1.621335868
cg10137837	0.002072286	4.31E-33	0.001994499	1.51E-08	1.039000627
cg06784991	0.001629848	1.15E-32	0.002473816	1.25E-22	0.658839735
cg25410668	0.002438088	1.28E-32	0.002840681	5.97E-34	0.858276119
cg26161329	0.001482569	2.01E-32	0.001544604	1.13E-15	0.959837669
cg11220950	0.001427245	8.01E-32	0.002101178	1.80E-21	0.679259698
cg24430580	0.000843139	9.95E-32	0.000947311	4.32E-17	0.890034131
cg24724428	0.002539763	1.82E-31	0.003078433	5.37E-21	0.825017941
cg19344626	-0.004609419	1.85E-31	-0.003295368	3.89E-14	1.398757211
cg04266460	0.001448352	2.14E-30	0.001119113	4.14E-15	1.294197045
cg17110586	0.001421701	3.45E-30	0.001860317	2.75E-23	0.764224733
cg14674720	0.001324651	4.76E-30	0.001831017	1.30E-20	0.723450731
cg10778288	0.000694576	8.01E-30	0.000580804	3.46E-15	1.195886777
cg17760405	0.000626231	9.56E-30	0.000802966	0.00013	0.779896525
cg20294304	0.002913236	1.25E-29	0.002490846	1.22E-13	1.169576777
cg04400972	0.001320183	1.51E-29	0.001785925	1.85E-19	0.7392152
cg04875128	0.004402121	2.32E-29	0.004203694	2.30E-29	1.047203078
cg19863655	0.00159659	2.52E-29	NA	NA	#VALUE!
cg05308819	-0.001527398	3.45E-29	-0.001615308	6.92E-22	0.945577074
cg21572722	0.001618051	4.84E-29	0.00263524	3.58E-38	0.614005278
cg25334393	0.001751469	5.43E-29	NA	NA	#VALUE!
cg11084334	0.001674349	7.61E-29	0.001728016	1.06E-41	0.968942833
cg03646916	0.002357435	8.90E-29	NA	NA	#VALUE!

cg15341124	0.001129609	1.01E-28	0.001580715	1.24E-13	0.714619289
cg06782035	0.001639252	1.01E-28	0.000688631	1.69E-06	2.380452014
cg01620164	-0.008987049	1.01E-28	-0.008097007	3.19E-29	1.109922402
cg20818778	0.000957245	2.06E-28	0.001091358	4.13E-13	0.877113681
cg10149533	-0.00217812	2.67E-28	NA	NA	#VALUE!
cg19784428	-0.003310308	2.71E-28	-0.00240413	1.54E-13	1.376925964
cg12317815	-0.002294044	3.90E-28	NA	NA	#VALUE!
cg03530962	-0.001571763	4.18E-28	-0.001510203	6.94E-18	1.040762812
cg02391713	0.001462429	5.19E-28	0.001591046	3.18E-26	0.91916216
cg18343474	0.000878024	8.37E-28	0.000956484	1.63E-18	0.917970541
cg22353329	0.001172574	8.61E-28	0.001501414	6.61E-19	0.780980146
cg06639320	0.002330505	8.76E-28	0.002970537	5.11E-19	0.784539921
cg07955995	0.001147601	1.87E-27	0.000622242	1.21E-08	1.844299116
cg08090640	-0.001388672	3.02E-27	-0.001048388	3.00E-07	1.3245783
cg20591472	0.001290985	3.28E-27	0.001883274	6.99E-12	0.685500135
cg03074925	0.000801183	3.31E-27	0.001028959	4.22E-11	0.778634878
cg18064714	0.001222424	5.11E-27	0.001698774	4.34E-20	0.719592177
cg10804656	0.002897793	5.41E-27	0.002995618	5.97E-17	0.967343717
cg25090514	0.00107206	8.74E-27	0.000759902	1.92E-12	1.410787956
cg05584950	-0.001054214	8.81E-27	-0.001236306	4.32E-09	0.85271239
cg03440125	0.001857009	2.07E-26	0.001940062	1.30E-08	0.957190639
cg18473521	0.003418834	2.17E-26	NA	NA	#VALUE!
cg00329615	-0.004826972	3.21E-26	-0.004851243	4.87E-27	0.99499707
cg05412028	-0.00176297	4.86E-26	-0.002304438	1.30E-17	0.765032455
cg09643544	0.000604387	5.37E-26	0.001103024	4.95E-11	0.547936119
cg25478614	0.001501238	5.95E-26	0.001647758	2.65E-14	0.911079298
cg15557036	0.001240044	6.08E-26	0.001483621	5.39E-14	0.835823095
cg22059812	0.000857447	7.24E-26	0.001409862	7.04E-14	0.608178139
cg02287710	0.001486127	7.98E-26	0.002009661	8.67E-15	0.739491451
cg21159778	0.002122983	8.10E-26	0.002375434	2.32E-19	0.893724525
cg08160331	0.002097476	8.14E-26	NA	NA	#VALUE!
cg21809447	0.000621675	9.88E-26	0.000770927	2.87E-08	0.806399105
cg06492796	-0.001705477	1.02E-25	-0.00095981	5.30E-06	1.776890326
cg23078123	-0.002161932	1.08E-25	-0.001863845	3.09E-26	1.159931538
cg23500537	0.001937876	1.19E-25	0.002226789	6.05E-19	0.870255693
cg26628907	-0.001597801	1.43E-25	-0.001744319	9.64E-17	0.916002873
cg24369989	0.001608894	1.71E-25	0.001672682	4.46E-10	0.961864891
cg05991454	0.001817133	2.07E-25	0.001822075	5.64E-18	0.997288004
cg24575083	0.000821755	2.15E-25	0.001239411	0.00377	0.663020842
cg07547549	0.001881939	2.53E-25	0.002577787	2.83E-39	0.730060075
cg22620090	0.001339591	3.15E-25	0.000436098	7.99E-06	3.071768746
cg08957484	0.002637604	3.62E-25	0.002481226	6.08E-18	1.063024478
cg03607117	0.001004499	5.18E-25	0.000922776	2.30E-17	1.088562095
cg13806741	0.001387567	5.26E-25	0.001858217	6.94E-18	0.746719567
cg17885226	0.001781157	5.27E-25	0.001945925	2.72E-10	0.915326482
cg21867345	0.001293886	1.16E-24	0.001565578	1.06E-10	0.826458677
cg20249566	-0.003498521	1.23E-24	-0.002876463	9.22E-14	1.216257938

cg24891133	0.001639269	1.43E-24	0.001432986	1.74E-09	1.143953049
cg23077820	0.001438267	1.74E-24	0.001451517	2.22E-10	0.990871941
cg11071401	0.001762615	1.89E-24	0.0021864	3.79E-30	0.806172317
cg22358580	0.000915972	2.64E-24	0.001330991	3.27E-13	0.688188237
cg12852499	0.000605865	3.44E-24	0.001211803	7.59E-12	0.499969684
cg19470159	0.000413678	3.46E-24	0.000857233	6.44E-08	0.482573288
cg09401099	0.001678956	4.64E-24	0.002029457	3.36E-21	0.827293231
cg01974375	-0.001217891	7.54E-24	-0.001518651	3.51E-19	0.801955709
cg21790626	0.0009704	7.90E-24	0.000631343	2.31E-05	1.537040365
cg04295144	0.004285485	9.90E-24	0.004404946	3.14E-44	0.972880297
cg09636661	-0.001685084	1.11E-23	-0.001774273	1.66E-13	0.949731782
cg17430979	-0.002422679	1.31E-23	-0.002503325	4.60E-11	0.967784668
