# Supplementary material for: The effect of age on DNA methylation in whole blood among Bangladeshi men and women
Source: BMC Genomics. 2019 Sep 10;20:704. doi: 10.1186/s12864-019-6039-9 (PMC6734473; doi:10.1186/s12864-019-6039-9)
Supplement: Supplementary file 7 — Number of top 91 age-associated CpGs in common between different RefFreeEWAS models using original dataset and a) top 100 age-associated CpGs in Validation dataset or b) significant (< 5e-8) age-associated CpGs in the Validation dataset (PDF 69 kb) [file 12864_2019_6039_MOESM7_ESM.pdf]

Additional File Table 7. Number of top 91 age-associated CpGs in common between different RefFreeEWAS models using original dataset and a) top 100 age-associated CpGs in Validation dataset or b) significant (< 5e-8) age-associated CpGs in the Validation dataset

	a)	b)
Ref_all*	24	95
Ref_men#	18	58
Ref_women#	19	81
Ref_all_sm^	25	92
Ref_men_sm~	18	69
Ref_women_sm~	22	83

* Model factors include sex and age

Model factors include age

^ Model factors include age, sex, and smoking

~ Model factors include age and smoking
