# Supplementary material for: The effect of age on DNA methylation in whole blood among Bangladeshi men and women
Source: BMC Genomics. 2019 Sep 10;20:704. doi: 10.1186/s12864-019-6039-9 (PMC6734473; doi:10.1186/s12864-019-6039-9)
Supplement: Supplementary file 8 — UCSC genome location information for a subset of the top 100 age-associated CpGs observed to be significantly associated with its Illumina assigned gene expression (PDF 112 kb) [file 12864_2019_6039_MOESM8_ESM.pdf]

Additional File 8. UCSC genome location information for a subset of the top 100 age-associated CpGs observed to be significantly associated with its Illumina assigned gene expression

> ###Table info. - cpg by expression among males

ID	UCSC_RefGene_Name	UCSC_RefGene_Group	Relation_to_UCSC_CpG_Island
cg09809672	EDARADD	TSS1500;5'UTR;1stExon	N_Shore
cg12934382	GRM2	1stExon;5'UTR	Island
cg24466241	FAM159A	Body	Island

> ###Table info. - cpg by expression among females

ID	UCSC_RefGene_Name	UCSC_RefGene_Group	Relation_to_UCSC_CpG_Island
cg03646916	ZNF197	TSS200	Island
cg08160331	MIR326	1stExon	Island
cg09636661	NFAT6	1stExon;5'UTR	Island
cg09643544	ZNF266	1stExon;5'UTR	Island
cg15557036	KAT7		Island
cg18473521	HOXC4	Body	S_Shore
cg21790626	ZNF671, ZIK1, ZNF814	5'UTR;1stExon	Island
cg21867345	ZNF197	TSS200	Island
cg25334393	RPL8	Body	Island

> ###Table info. - age interaction

ID	UCSC_RefGene_Name	UCSC_RefGene_Group	Relation_to_UCSC_CpG_Island
Males			
cg09809672	EDARADD;EDARADD;EDARADD	TSS1500;5'UTR;1stExon	N_Shore
Females			
cg03646916	ZNF197	TSS200	Island
cg08160331	MIR326	1stExon	Island
cg21867345	ZNF197	TSS200	Island
